# A Novel Method for Determining the Contact Pattern Area in Gear Meshing Based on Computer Processing of Pressure Measurement Film Images

**DOI:** 10.3390/ma18143230

**Published:** 2025-07-08

**Authors:** Paweł Fudali, Patrycja Ewa Jagiełowicz, Adam Kalina, Piotr Połowniak, Mariusz Sobolak, Waldemar Witkowski

**Affiliations:** The Faculty of Mechanical Engineering and Aeronautics, Rzeszow University of Technology, 35-959 Rzeszów, Poland; pfudali@prz.edu.pl (P.F.); pejagielowicz@prz.edu.pl (P.E.J.); piotrp@prz.edu.pl (P.P.); msobolak@prz.edu.pl (M.S.); wwitkowski@prz.edu.pl (W.W.)

**Keywords:** gear tooth contact, contact area measurements, experimental tooth contact analysis, two-sheet type pressure film, mechanical engineering, computer image processing

## Abstract

The contact pattern between gear teeth is one of the most significant indicators of proper gear operation. This paper presents an analysis of the contact pattern of gears with a sinusoidal profile. The gear geometry was obtained through direct solid simulation of the machining process. Generally, analytical, numerical, and experimental methods are used for contact pattern analysis in gearboxes. This article presents contact pattern investigations using numerical methods and a novel experimental method that utilizes pressure measurement films. A proprietary program using image analysis was used for the contact pattern analysis. The numerical studies utilized the Finite Element Method (FEM) and the CAD method. The results obtained from the presented methods show good convergence.

## 1. Introduction

Accurate determination of the tooth contact pattern is paramount in gear analysis, as it directly relates to a gear’s ability to transmit rotary motion effectively. Theoretically, perfect gears, also known as exact gears, precisely transmit rotary motion according to a preset function and typically exhibit linear tooth contact. However, gearboxes with linear tooth contact are highly sensitive to assembly errors. In contrast, gears with point contact generally offer lower strength compared to those with linear tooth contact [1].

At any given moment, several pairs of surfaces may be in contact, and the sum of these instantaneous contact patterns produces the total contact pattern. The size and position of instantaneous contact patterns are critical indicators of the correct gearing. Their shape is affected by the tooth profile, the tooth line, the elastic deformation of mating surfaces, the gearing accuracy, the assembly, and the tool marks. Modifying the profile of one or both gears can alter the size of the contact pattern. Determining the instantaneous contact pattern provides a preliminary assessment of the gear design’s correctness. Its shape, position, and changes during gear rotation dictate the gearbox’s operational smoothness and load-carrying capacity. The determination of the contact pattern is most often performed for unloaded or lightly loaded gears and is crucial for gear dynamics and tooth contact strength calculations [1,2,3,4,5,6].

There are three main types of methods to determine the contact pattern: analytical methods, numerical methods (utilizing the Finite Element Method, and Boolean methods based on CAD system modeling techniques), and experimental methods (primarily involving direct observation, indirect ‘freezing’ deformation methods). Analytical methods require specific equations to describe the tool and machined gear surfaces, as well as their geometric and kinematic relationships. Homogeneous matrices of transformation are commonly used in the geometric analysis of gears to describe the simultaneous rotation and translation of the coordinate system. The Finite Element Method (FEM) allows for solving the non-linear problems inherent in gears, modeling complex contact conditions, including elastic–plastic material properties and tooth geometry [7]. Mathematical methods supported by computational techniques are increasingly used in gear geometry determination [8,9,10,11,12]. Works [13,14] present extended solutions combining kinematic and FEM analysis to determine meshing non-uniformity. Studies often focus on contact patterns with an elliptical shape [15,16]. Numerical methods generate surfaces as discrete sets of points [17,18,19]. Authors often compare contact patterns obtained via Litvin’s kinematic method and other computer software with results from the finite element method [20,21,22,23]. FEA is predominantly used for novel or unconventional gear solutions. A significant challenge in applying numerical methods to gear theory is developing suitable software. Leading machine tool manufacturers (e.g., Gleason, Klingelnberg-Gruppe) possess relevant software, but it is proprietary and distributed under license [24,25]. CAD systems are also used for mesh analysis and geometric contact pattern determination, as well as for gear machining for various tooth types [10,26,27,28,29]. Studies [30,31] have used discrete surface synthesis methods to determine the contact pattern. CAD techniques are also applied to gear modification issues and to compare the results of numerical calculations [31]. In industrial settings, the mating contact area during operation is typically determined experimentally, often by placing the gears on a specialized test stand and applying the marking compound method. Experimental methods are successfully applied to models created via Rapid Prototyping techniques. For instance, stereolithography models, though dimensionally accurate, are typically made of low-strength resin, limiting their use to demonstration or static testing based on the model similarity principle. Experimental studies often leverage model similarity. Contact pattern testing on a test bench can be conducted directly and indirectly. A direct observation of the contact pattern is possible as a result of the transparency of the gear material. Studies indicate that wetting the model surfaces with liquid facilitates observation [32,33,34]. This direct method provides a preliminary assessment of the accuracy of the gear design and information on the contact pattern shape and position of the contact pattern. However, a precise size determination is problematic, requiring a 3D scan with surface color differentiation (texture). Therefore, this method is restricted to transparent plastics, limiting its broader applicability. An indirect observation method is the vacuum casting technique, which involves pouring silicone into a container with the deformed gear under static loading. This process fixes the deformation of the loaded model, preserving the contact pattern in silicone in its exact loaded shape. The resulting shape reflects the instantaneous contact pattern between the mating teeth. Contact patterns obtained in silicone typically have a flake-shaped structure, allowing their approximate surface determination, which is sufficiently accurate for comparison using a 2D scanner [35].

Measuring the contact area between gear teeth using a thin-film sensor requires a specialized sensor design due to the small contact area of gear tooth surfaces. Thin-film sensors are applicable across industries for measuring contact areas between components [36,37,38]. Furthermore, thin-film sensor systems can be integrated directly into tools or component surfaces during production for real-time monitoring of force, temperature, and their distributions with high spatial resolution, facilitating condition monitoring and predictive maintenance [39,40,41,42,43,44]. The advantages of thin-film sensors for measuring contact areas include high precision, simplicity, and accurate dynamic pressure measurement. They provide a non-contact measuring method, reducing system errors and random errors while offering strong anti-interference capability and high precision. Additionally, gel thin-film array capacitor sensors can accurately measure total dynamic occlusion with high flexibility and resolution, ensuring accurate and repeatable measurements [45].

Pressure measurement films (e.g., Prescale by FujiFilm or Surface Profiler Film by Sensorprod) are widely used in medical research [46,47,48,49,50,51], mechanical engineering [52,53,54,55], and geology [56,57]. These films allow for the determination of contact area, average pressure, maximum pressure, and pressure distribution. However, they are limited to static loads. For dynamic contact or pressures exceeding 300 MPa, alternative pressure measurement methods should be used [58]. Also, the film scanning must be performed according to the manufacturer’s specifications. The scan delay after measurement can cause incorrect results [59].

This article presents a novel experimental method for measuring the contact pattern using pressure measurement films, serving as an effective complement and verification of theoretical studies. Experimental investigations (LTCA) combined with the analysis of results obtained through theoretical methods (TCA in CAD and LTCA using FEM) have demonstrated the high consistency and practical utility of the developed approach. This method not only confirms the agreement of the results obtained from the pressure films with those from the numerical and analytical studies, but also indicates its applicability in workshop conditions for quick assessment of the correctness of the gear assembly. Additionally, the algorithm developed, utilizing a common office optical scanner, offers an economical alternative to specialized equipment, opening new perspectives in quality monitoring and diagnostics of cylindrical gears, including those with a sinusoidal profile.

## 2. Materials and Methods

### 2.1. Theoretical Basics

#### 2.1.1. General Information of Gear Surface Modeling in CAD System

**Figure 1:**
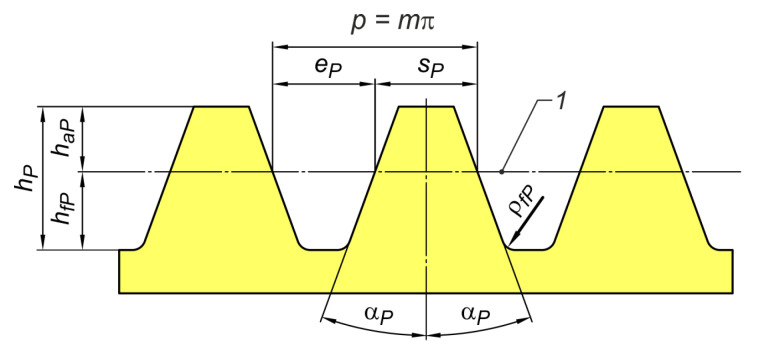
Standard basic rack profile for involute gears: 1—datum line, p—pitch, m—module, $e_{p}$—space width, $s_{p}$—tooth thickness, $h_{p}$—tooth depth, $h_{aP}$—addendum, $h_{fP}$—dedendum, $\alpha_{p}$—pressure angle, $\rho_{fP}$—fillet radius.

Figure 1 shows a standard basic rack profile for involute gears. The tooth profile normal section through the teeth of a basic rack corresponds to an external gear with the number of teeth z → ∞ and the diameter d → ∞. It is a linear profile [60,61].

**Figure 2:**
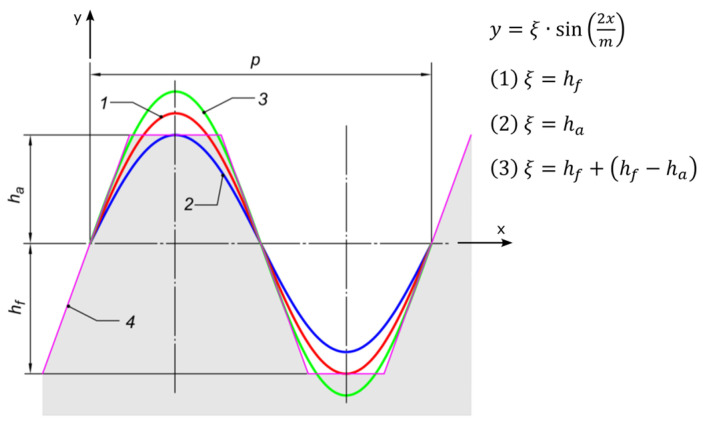
Construction of a sinusoidal reference profile: 1—base profile, 2—low profile, 3—high profile, 4—straight-line profile (for involute gears).

The outline shown in Figure 1 is the reference profile, in which on the datum line, the tooth thickness is equal to the space width. Figure 2 shows the sinusoidal profile (1, 2, 3) against the background of the straight outline (4). This profile, which is a representation of the trigonometric sine function, should be symmetric with respect to the datum line. It can be ‘zero’ (1), low (2), or high (3). Symmetry is due to the need to obtain a coupled outline of the mating gears.

**Figure 3:**
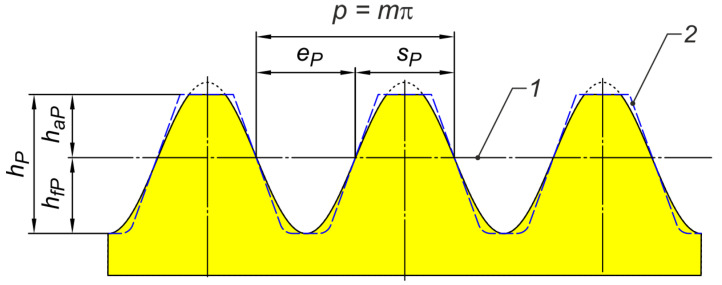
Assumed reference profile for sinusoidal gears: 1—datum line, 2—straight-line profile (for involute gears).

Figure 3 shows the sinusoidal reference profile assumed for the study. It is the ‘zero’ profile of the sinusoidal curve. In this case, the apex of the sinusoid is the root of the tooth space.

**Figure 4:**
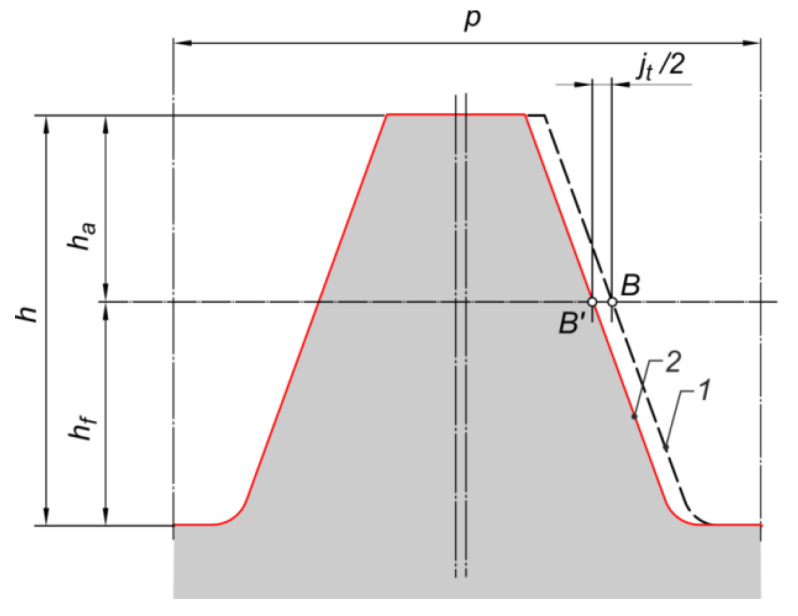
Correction of the reference profile—introduction of circumferential clearance for involute gears: 1—profile before correction, 2—profile after correction.

**Figure 5:**
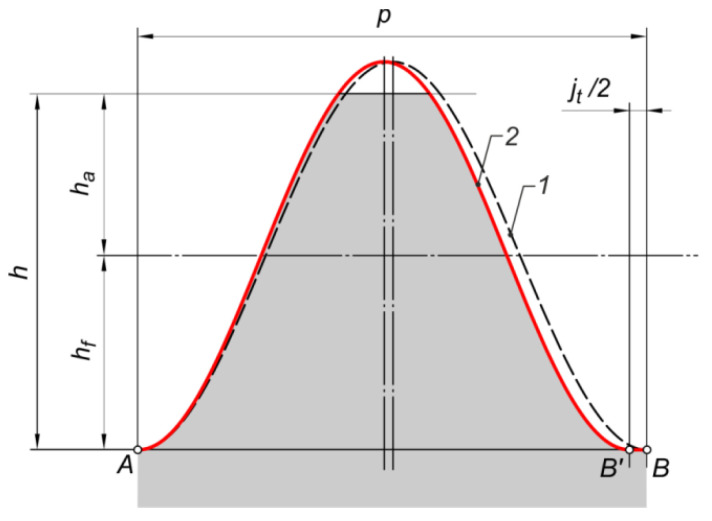
Correction of the reference profile—introduction of circumferential clearance for sinusoidal gears: 1—profile before correction, 2—profile after correction.

Due to the need to use clearance, the reference profile was modified. Figure 4 shows the idea of the reference profile correction for involute gears. In order to obtain the circumferential clearance $j_{t}=0.05m_{n}$, a scaling of the profile sinusoid was made. Figure 5 shows how to correct the gears with a sinusoidal profile. The ‘width’ of the sinusoid was scaled by a factor f, designated as $f=\frac{\left(p-\frac{j_{t}}{2}\right)}{p}$.

**Figure 6:**
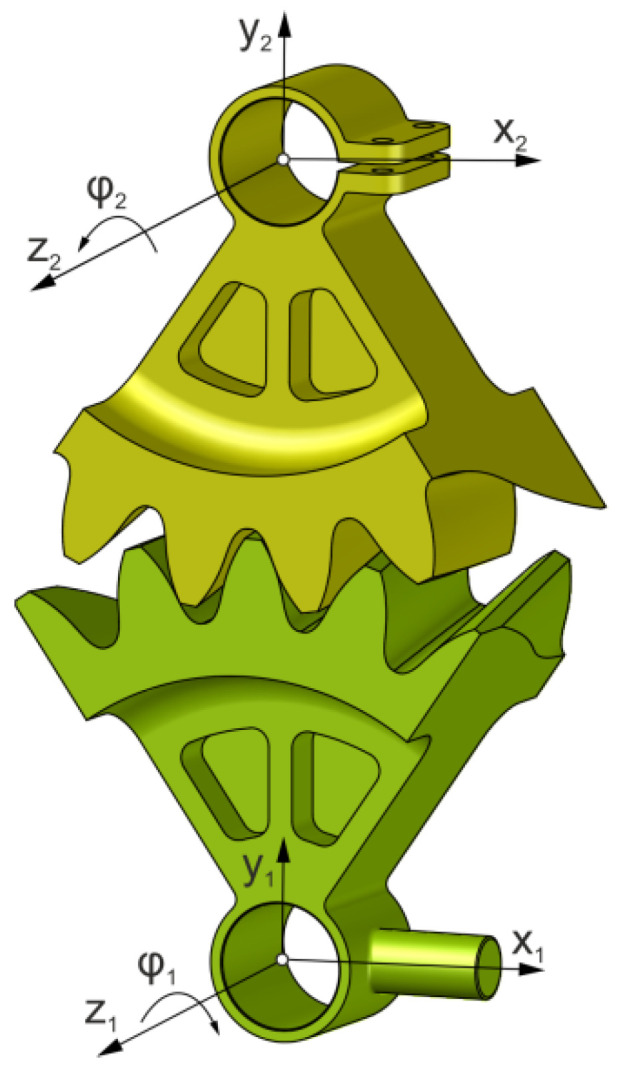
Overview of the gearbox CAD models.

Based on the reference profiles, solid 3D models of the gears were created in the CAD environment (Figure 6). A direct solid CAD method was used to simulate machining [62]. The parameters of the gears are given in Table 1.

#### 2.1.2. Determination of Contact Pattern in CAD Environment

Solid CAD models of the gears were prepared for assembly on the test stand. They will also be used to produce actual models. To determine the contact pattern in the CAD environment, the gear models are assembled into a gearbox, as shown in Figure 6.

The moving wheel model is rotated at such an angle with respect to the z_1_-axis that it is intersected on the side of the other wheel for a given value $\delta_{w}$. This value can correspond to the thickness of the oil film or the thickness of the marking compound. The value of the intersection on the pitch diameter was assumed to be equal to 15 µm [8,9,32,63]. Simulating the operation of a gearbox, the models rotate with a fixed discrete step according to the gear ratio. At a given position of the transmission elements, the common part of the models is determined.

#### 2.1.3. FEM Analysis

**Figure 7:**
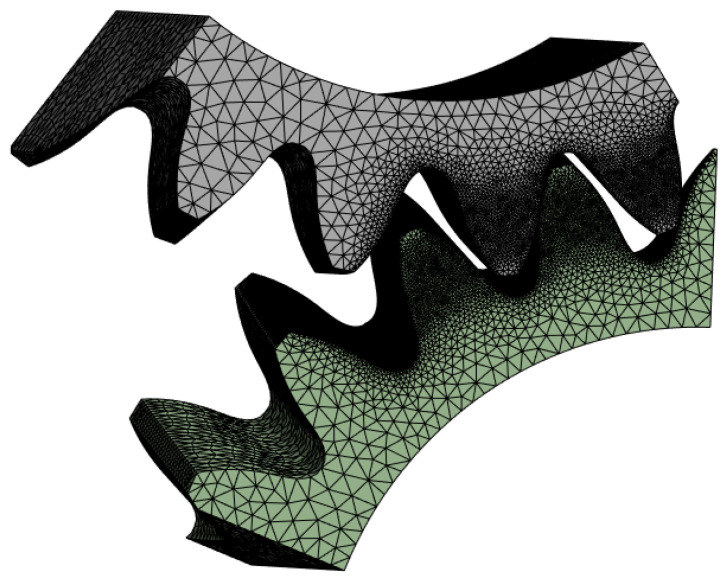
Mesh on the gear model.

One of the methods to obtain the contact pattern is numerical analysis using FEM. Numerical methods are successfully used to simulate complex engineering problems, as shown in the works [11,17,64]. As part of the research work, a study of the mating of the gear was carried out using this method in the ANSYS 2024 R1 software (Ansys, Inc., Canonsburg, PA, USA). The continuum model of the gears was divided into finite elements. Due to geometric complexity, second-order tetrahedral elements were used. The key resulting parameter was the aspect of contact between the surfaces on the side of the tooth, so the mesh was compacted in this area. The computational model consisted of about 3.15 million elements (Figure 7). The gearbox was set at the same position as in the CAD and the experimental analysis. A load torque of 46 Nm was assumed. A static analysis was performed, taking into account the frictionless contact between the sides of the teeth. During the analysis of the contact pattern, friction forces are negligible compared to other forces in the system [7,8,10]. The calculation resulted in a contact area, defined as the area where the distance between the walls did not exceed 15 µm—analogous to the study using the CAD method. The contact area adopted in this way made it possible to reproduce the conditions that prevailed in the experiment.

#### 2.1.4. Pressure Measurement by Using Two-Sheet Type Film

The study was carried out using two sets of two-sheet type pressure measurement films developed by different manufacturers. These are Prescale Super Low Pressure (LLW) from FujiFilm (Fujifilm Europe GmbH, Ratingen, Germany) and Surface Profiler Film (SPF) from Sensorprod (Sensor Products Inc., Madison, NJ, USA). The selected parameters of the films used are shown in Table 2 and Table 3.

**Figure 8:**
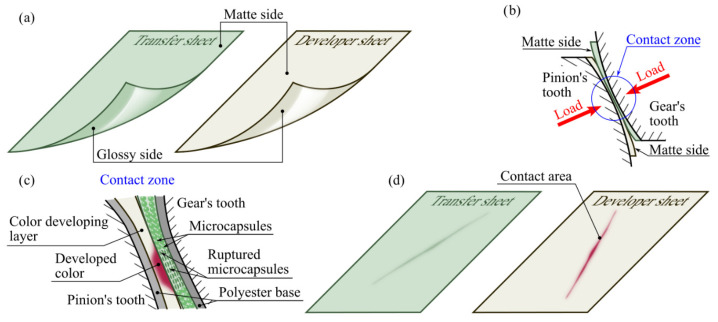
Sensroprod SPF-D film set: (**a**) transmitter sheet (left) and developer sheet (right) before loading, (**b**) mutual alignment of sheets for measurement, (**c**) release of active substance from transmitter in contact zone, (**d**) transfer sheet (**left**) and developer sheet (right) after completion of test measurement.

Both sets consist of two types of film, i.e., a sheet that acts as a transmitter (transfer sheet) and a developer sheet (Figure 8a). The transmitter contains microcapsules with a special active substance, whereas the developer sheet (receiver) has a layer whose role is to develop the color representing the pressure distributions in the contact zone. Discoloration of the receiver occurs due to the active substance from the transmitter’s receiver capsules. To accomplish this, the properly cut sheets of the two films must be folded together so that they are facing each other with their matte sides when measured (Figure 8b). Placing the foil between the contacting sides of the gear wheel teeth and then loading it will cause pressure to build up in the contact zone. The resulting pressure leads to the rupture of the capsules and the release of the active substance in the contact zone (Figure 8c). The duration of the load application and the time required to develop the color on the receiver sheet depend on the recommendations provided by the specific manufacturer. The shape of the contact zone and the distribution of stresses in it are determined by the receiver sheet, while the transmitter sheet can be discarded (Figure 8d).

#### 2.1.5. Image Processing—Custom Program for Sample Image Analysis

Main assumptions

A program was developed to accurately determine the area of the docking trace and automate this process. The programs were written in a MATLAB environment. The main assumptions of the prepared program are as follows:

- Automation—the program allows the analysis of multiple samples, with limited user participation. The results of the obtained analysis are automatically saved and catalogued. The role of the user is reduced to the appropriate saving of file names in the working directory of the program, as well as determining the type and key parameters of the analyses performed.
- Modularity—the main program uses specialized subprograms (functions). Each function is used to perform a different type of analysis or perform a specific task. The functions can work independently.

Description of the functionality of main program and selected subprograms

The main program includes instructions related to the recognition of files for analysis, which are placed in the working directory. In addition, it communicates with the user to obtain key parameters for analysis. An example of such a parameter is the *Sensitivity* parameter, which determines the threshold of detecting whether a given image pixel can be classified as part of the contact area or not. Subsequently, these parameters and the data extracted from the images are passed to separate subprograms that perform specific functions. The program also performs an aggregate representation of the selected analysis results. The obtained results are saved in the data space of the MATLAB program and on the user’s disk in the form of data tables, graphs, and processed images.

The program implements a system to recognize the names of files contained in the program working directory. Its path can be freely specified in the program (this is responsible for the Path variable, the default value of which is the Samples directory located in the working directory of the MATLAB R2023b program (MathWorks, Inc., Natick, Massachusetts, USA), where the written program files are located). In order to ensure correct operation of the program, all image files with scanned samples should be saved in PNG format and moved to the Samples directory. In addition, the following syntax for the image file name should be used, along with an extension specifying the file type:

124a−140_5.png

where the different colors stand for the following:

- **red**: alphanumeric identification of the sample (it is recommended to use the number itself),
- **purple**: the actual length of the sample in the horizontal direction, expressed in whole numbers; the length is stated in millimeters,
- **blue**: hundreds of the horizontal length of the sample; if the length of the sample can be expressed by an integer, this filename can be omitted,
- **green**: file extension (by default in Microsoft Windows, this part of the file name is invisible),
- **black**: separators of particular parts of the name.

Accordingly, the file named 124a-140_5.png will be interpreted by the program as sample No. 124a with a length of 140.5 mm, which is saved as an image in PNG format.

The measurement of the contact area is implemented through a subroutine (hereafter referred to as the function) *ContactArea*. The *ContactArea* function returns the value of the measured contact area *MCA*, expressed in square millimeters, and a monochrome image, hereafter called *ContactMap*. In this image, the black pixels mark the areas that the program has classified as those in which contact has occurred.

The arguments of the program are the following variables:

- *Image*—a file with a color or black-and-white scan of the sample. NOTE: the image should first undergo preprocessing to crop the samples and remove unwanted noise and scanning artifacts,
- *l_H*—horizontal length of the sample in mm,
- *l_V*—vertical length of the sample in mm,
- *Sensitivity*—sensitivity expressed in bits, ranging from 0 to 255,
- *Drawings*—control variable taking the value of 0 or 1. The value of 0 means that the function will not display graphs and drawings generated after analyzing a single image, while 1 means that all drawings and graphs will be displayed. When analyzing a large number of files, when this program is called in a loop, it is recommended that this variable take the value 0.

**Figure 9:**
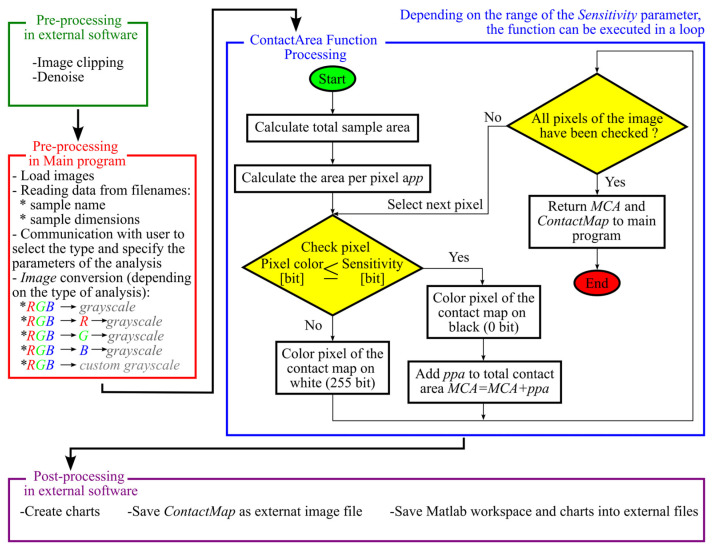
Simplified algorithm for the image analysis process using the *ContactArea* function with preprocessing and postprocessing.

Figure 9 shows a simplified algorithm for the *ContactArea* function. The following algorithm also takes into account the elements of preprocessing, which is implemented in the main program.

A code fragment of the *ContactArea* function responsible for detecting contact pixels and generating a contact map is presented in Appendix A.

The algorithm shown in Figure 9 involves two-stage preprocessing, that is, preprocessing performed by the user using external graphics software. As part of this stage, the image obtained by scanning the sample is cropped and cleaned of noise, artifacts, and unwanted contaminants that may have been created by touching the sample.

**Figure 10:**
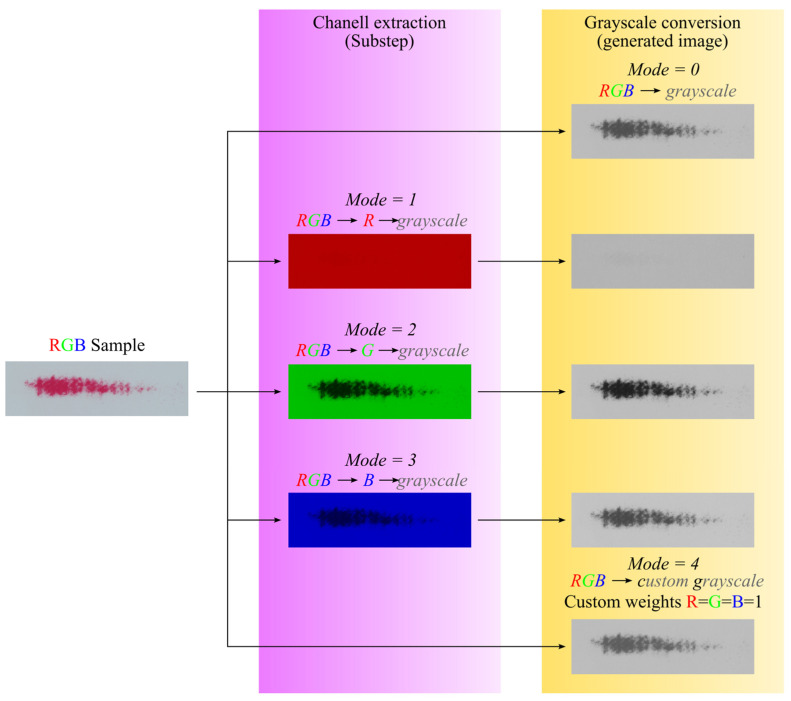
Images generated by the *rgbExCon()* function. NOTE: The images obtained in the Chanell extraction substep are not returned by the *rgbExCon()* function, as they should not be passed to the *ContactArea()* function.

On the other hand, the preprocessing performed in the written program, in addition to the steps described earlier (extracting sample information based on their names), also includes conversion of the loaded sample image. The default conversion involves converting a color image stored in the RGB system into a grayscale image. MATLAB’s built-in function *rgb2gray()* was used for this. This function calculates the color of a grayscale pixel as a weighted average of the values of the individual components of the primary colors. The weights of the RGB color components are 0.2989, 0.5870, and 0.1140, respectively. The developed program also allows other types of image conversions, which are labeled *RGB → R → grayscale*, *RGB → G → grayscale*, and *RGB → B → grayscale* in the discussed algorithm. The images obtained as a result of these conversions allow the analysis of samples in such a way that only the selected color component of the sample is checked (the function returns a monochrome image in which the selected component is converted to a grayscale image). In contrast, the *RGB → R → custom* grayscale conversion calculates a pixel-scale color based on a weighted average in which the weights of the individual components are defined by the user. The *rgbExCon(Image,Mode)* function was developed to perform one of the above conversions. This program performs the conversion of the transferred image based on the *Mode* parameter—which determines the type of conversion. Once the conversion is complete, the *rgbExCon()* function returns a grayscale image. Figure 10 shows how the samples returned by the functions look depending on the type of conversion selected.

It is worth mentioning that the images obtained in the *Chanell extraction substep* (Figure 10) are not converted to grayscale using the *rgb2gray()* function. In this case, the value of the R, G, or B component is equal to the gray shade of the generated map. At this point, it should be added that the function *ContactArea()* recognizes whether the image sent to it is a color or monochrome image. In the case of an RGB image, the standard conversion characteristic of the *rgb2gray()* function is performed.

The main task of the *ContactArea()* function is to calculate the total area of the contact pattern. To accomplish this, the program first calculates, based on the dimensions of the sample transmitted to the program, what its total area is expressed in mm. This area is then related to the total number of pixels of the image sent to the program. As a result, the *ppa* parameter expressed in mm^2^/pixel is calculated.

The function checks all image pixels and determines whether the currently analyzed pixel can be qualified as a part of the contact area (Figure 9—the first conditional instruction). According to the presented algorithm, all pixels whose grayscale lightness is less than or equal to the lightness specified by the *Sensitivity* parameter are qualified as contact areas. The number of pixels positively identified, multiplied by the *ppa parameter (area per pixel)*, determines the size of the measured area of the *MCA (Measured Contact Area)* contact pattern.

**Figure 11:**
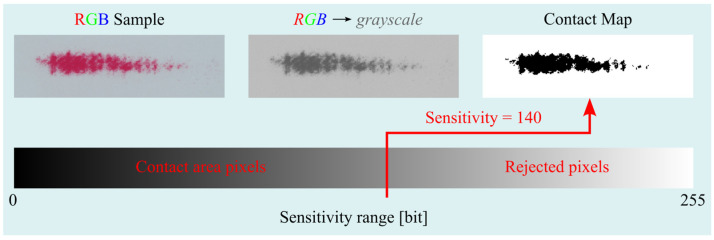
Generated contact map. NOTE: The function designed *ContactArea()* is for analyzing only one sample and for one value of the *Sensitivity* parameter. Therefore, in the main program, it was written in such a way that it is possible to analyze multiple samples for a certain range of the *Sensitivity* parameter. Then, the function is called *ContactArea()* in appropriately prepared loops, but the description of this part of the program is not important, and so it will be omitted.

The *ContactArea()* function also generates a contact *map (Contact Map)*. This is a monochrome image with a resolution equal to that of the image being analyzed. The black pixels of the contact map indicate the areas that the function considered as contact areas according to the first conditional instruction shown in Figure 9, while the white area of the map defines the sample area where the contact does not occur. An example of a contact map together is shown in Figure 11.

After completion of a given operation of the *ContactArea()* function, the main program saves the contact map as an image in PNG format to a special working directory whose name refers to the sample name. Due to the fact that this function can be called repeatedly for the same sample to check it for several values of the *Sensitivity* parameter, the files are named as follows:

sample_number−contact_map_for_sensitivity−139contact_area−areamm2.png

For example, for sample No. 16, analyzed for a *Sensitivity* parameter equal to 139, the measured area was 85.2661 mm^2^, and the contact map was saved as a file named:

Sample_16−contact_map_for_sensitivity−139contact_area−85.2661mm2.png

**Figure 12:**
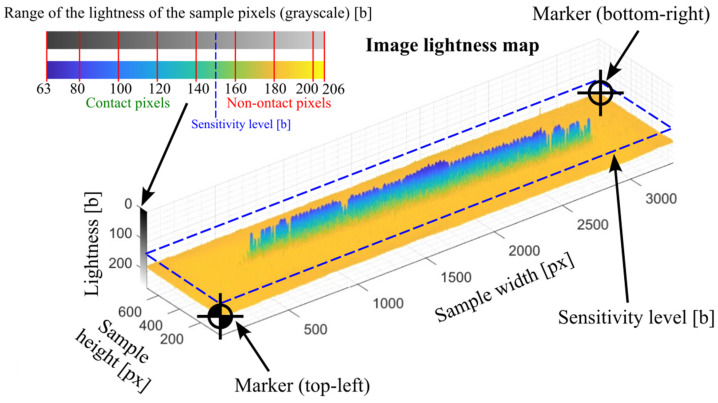
Pixel lightness map of the sample.

**Figure 13:**
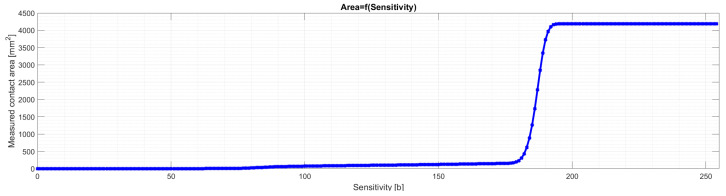
Effect of *Sensitivity* parameter on measured contact area.

After analyzing all samples for all *Sensitivity* values, the program saves the obtained results in the results folder and generates summary graphs. These are, in turn, a graph in the form of a 3D grid (Figure 12) and a graph showing what the relationship is between the *Sensitivity* parameter and the measured area of the contact area (Figure 13). For a better representation of the nature of the relationship between the *Sensitivity* parameter and the measured area of the contact trace, the points on the aforementioned graph are connected by straight-line segments.

**Figure 14:**
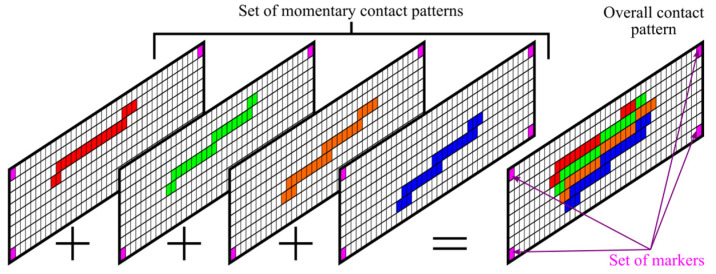
Overall contact pattern determined by the set of momentary contact patterns.

An important advantage of monochromatic contact maps is that images of this type can be easily processed with easy-to-use graphics editors. Using a suitable marker system or clipping mask and setting the alpha channel of white pixels to 0, it is possible to easily sum sets of contact maps. With momentary contact patterns determined for different positions of meshed gears, it is possible to determine the overall contact pattern (Figure 14).

An additional program was developed for a more complex analysis of momentary contact maps of momentary contact patterns. The developed program allows the following:

- the curve describing the upper outline line of the contact pattern $y_{U}\left(x\right)$,
- a curve describing the bottom outline line of the contact pattern $y_{B}\left(x\right)$,
- a curve describing the mean contact line $y_{M}\left(x\right)$,
- the user can determine the rate of the polynomial used to describe the above curves $P_{D}\left[u,v,w\right]$,
- the user can obtain the values of the coefficients standing at the individual expressions of the determined polynomials,
- determination of the angle of slope of the mean contact line to the x-axis (the horizontal edge of the sample) when using a polynomial of degree 1 $\alpha_{M}$,
- determine the length of the momentary contact pattern along the mean contact line $L_{MCL}$,
- determine the contact area of the contact pattern $A_{CCA}$ described by polynomial curves.

**Figure 15:**
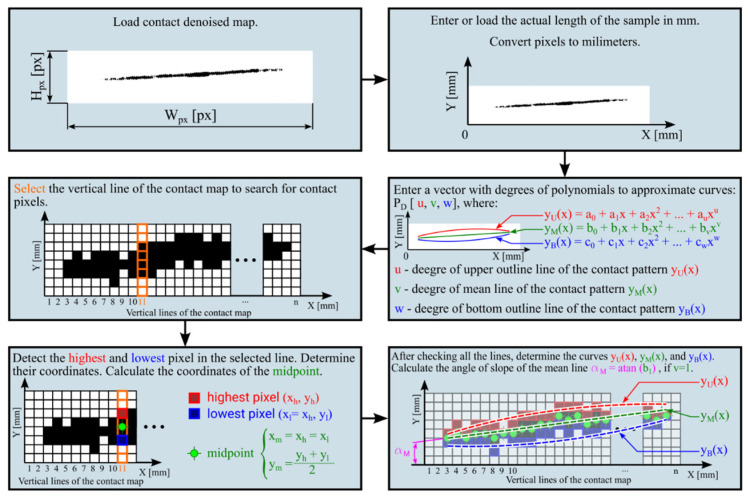
Simplified graphical algorithm of the contact maps analysis program.

**Figure 16:**
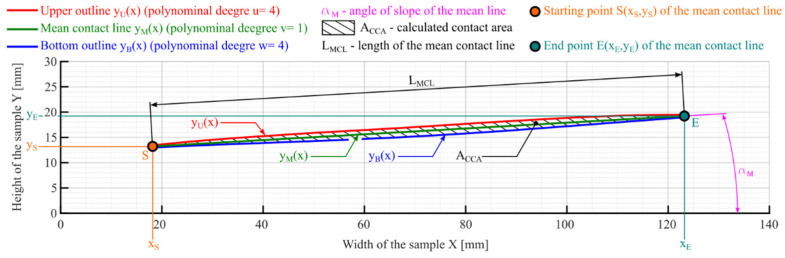
Examples of polynomial curves with a description of the most important parameters determined using the contact maps analysis program.

In Figure 15, a simplified graphical algorithm of the mentioned program is shown. It is highly recommended to denoise the contact map in external software or by using denoising algorithms before analysis. On the other hand, Figure 16 shows example curves determined from the contact map analysis.

As a result of analyzing the curves shown in Figure 16, it is possible, i.a., to accomplish the following:

- Determine the angle of slope of the mean contact line $\alpha_{M}$—for the mean contact line described as the line function $y_{M}\left(x\right)=b_{0}+b_{1}x$ (polynomial degree v=1):
  (1) $$\alpha_{M}=\mathrm{atan}\left(b_{1}\right).$$

- Determine the length of the momentary contact pattern along the mean contact line $L_{MCL}$ with Equation (2):

(2) $$L_{MCL}=\sqrt{{\left(x_{E}-x_{S}\right)}^{2}+{\left(y_{E}-y_{S}\right)}^{2}},$$

where

    - $x_{S}$ and $x_{E}$—horizontal coordinates of the first $\left(S\right)$ and last $\left(E\right)$ pixels/points of the momentary contact pattern, respectively;
    - $y_{S}$ and $y_{E}$—vertical coordinates of first $\left(S\right)$ and last $\left(E\right)$ pixels/points of the momentary contact pattern, respectively, calculated using the equation describing the mean line of the momentary contact pattern $y_{M}\left(x\right)$.

- Determine the contact area of the contact pattern $A_{CCA}$ described by the polynomial curves: $\;y_{U}\left(x\right)$—for upper outline and $y_{B}\left(x\right)$—for bottom outline:

(3) $$A_{CCA}=\int_{x_{S}}^{x_{E}}y_{U}\left(x\right)-\int_{x_{S}}^{x_{E}}y_{B}\left(x\right),\;A_{CCA}=\int_{x_{S}}^{x_{E}}a_{0}+a_{1}x+a_{2}x^{2}+\ldots +a_{u}x^{v}-\int_{x_{S}}^{x_{E}}c_{0}+c_{1}x+c_{2}x^{2}+\ldots +c_{w}x^{w}$$

where

    - u—degree of upper outline line of the contact pattern $y_{U}\left(x\right),$
    - w—degree of bottom outline line of the contact pattern $y_{B}\left(x\right).$

- After rotating all the curves by the angle $\alpha_{M}$, so that the mean line of the contact pattern is horizontal, it is possible to determine a function that allows for a convenient way of determining the change in the contact pattern width as a function of the contact line length.

### 2.2. Experimental Procedure

#### 2.2.1. Gear Manufacturing

The PolyJet printing method was used to manufacture gears with a novel tooth profile. For gear printing, the RGD720 material was used, and the RGD705 material was used to print the supports. The hatching distance was set at 16 [µm]. The material parameters are presented in Table 4. After printing on the Objet Eden 260 VS printing machine, the gears were washed in a high-pressure washer.

#### 2.2.2. Stand Description

**Figure 17:**
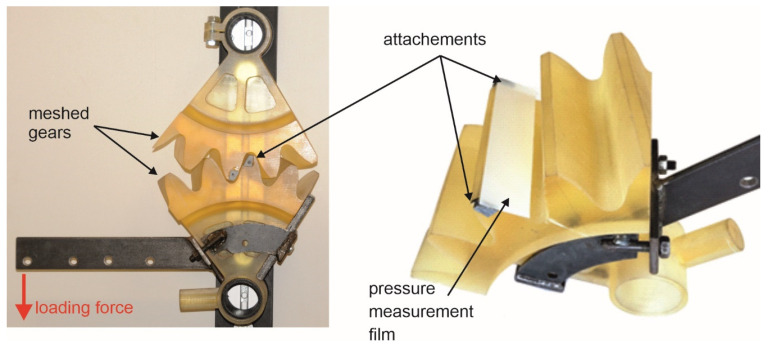
Rotational moment testing stand.

The experimental test stand is presented in Figure 17. The rotation of the upper gear was blocked. The lower gear was transferring the rational moment to the meshing gears. The rotational moment was obtained by the gravity force of the weights. The gray attachments, visible in the figure on the right, are used to attach the film in such a way as to minimize the impact of how the film is attached to the results of the experiment. In the case of polymer wheels, they can be attached using glue. For steel wheels, magnets can be used, so that the gear does not need to be cleaned after measurement. To accurately determine the length of the tooth side, special markers were applied to the developer sheet using a CD marker to determine the dimensions of the tooth side on the specimen scans.

#### 2.2.3. Determination of Gear Tooth Line and Surface Contact

For each gear meshing position, a transfer sheet and developer sheet were glued to gears separately (Figure 17). The adhesive area was outside the gear, so the adhesive layer thickening was not affected on the contact surface. After applying the rotational moment, for each gear arrangement, the pressure measurement time was 60 s—due to the manufacturer’s recommendation. The room temperature and humidity were also measured. The temperature was 23 °C, and the humidity was 57%. The sheet films were left in the same environment for 30 min. After this time, they were scanned on an Epson Perfection V600 Photo scanner (Seiko Epson Corporation, Owa, Suwa-shi Nagano-ken, Japan) with 300 dpi resolution.

## 3. Results and Discussion

### 3.1. CAD Analysis of Tooth Contact

**Figure 18:**
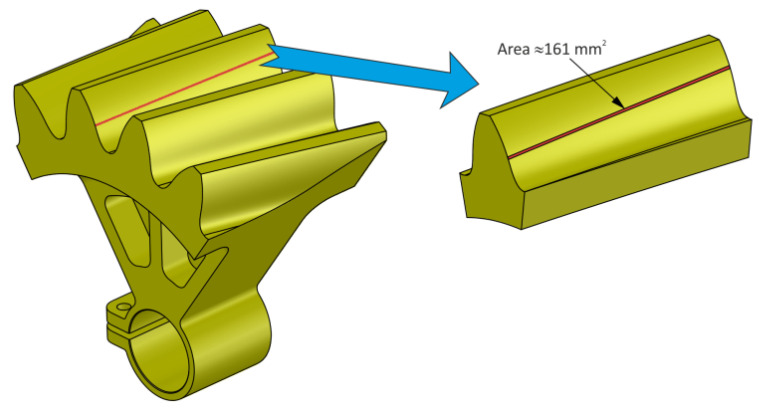
Contact pattern determined in the CAD environment.

Figure 18 shows the instantaneous contact pattern for the selected angular position of the gears. The area of the instantaneous contact pattern determined in the CAD environment is about 161 mm^2^. At the same position, the contact pattern will be analyzed experimentally and by FEM.

### 3.2. FEM Analysis of Tooth Contact

**Figure 19:**
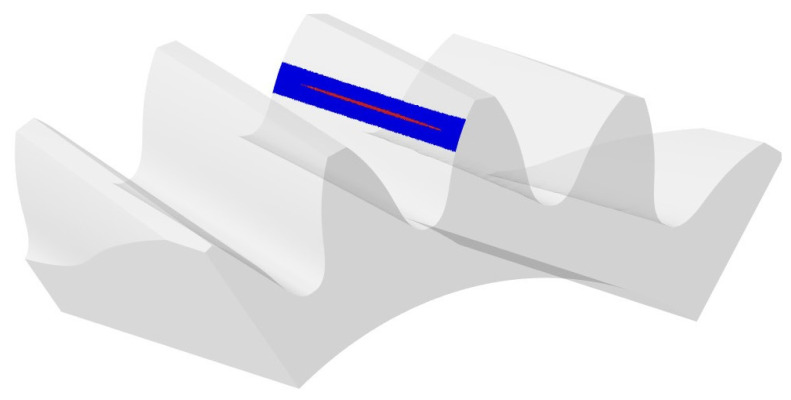
Contact pattern obtained from analysis using FEM. Blue rectangle represent analysed area and red area shows contact pattern.

Analysis of the results using FEM indicated significant similarities to the analysis using CAD. The contact pattern is located in a similar position, but unlike previous studies, it does not extend across the width of the tooth. Analyzing the contact zone, marked in red in Figure 19, its area of 137 mm^2^ can be determined.

Due to the achieved mesh convergence, the finite element size was reduced until further reduction did not result in a significant increase in the accuracy of the results but increased the calculation time.

### 3.3. Image Data Processing

#### 3.3.1. Image Data Processing—Contact Area Measurement

**Figure 20:**
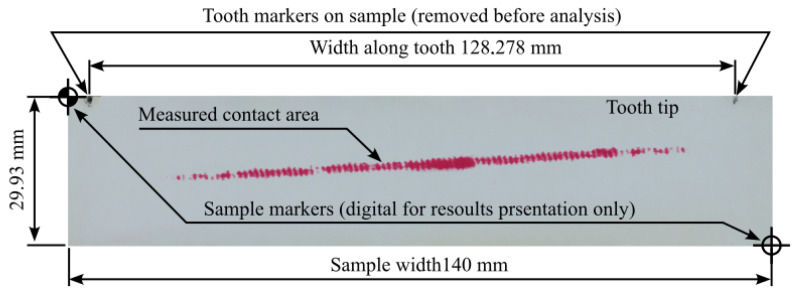
Sample 01Y-140_ before preprocessing with description of key parameters and determinations.

**Figure 21:**
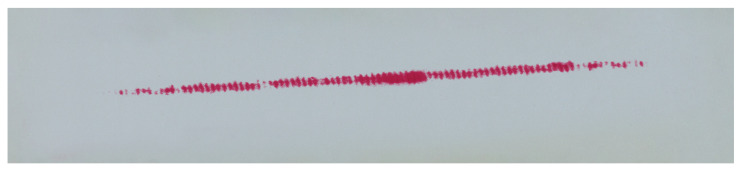
Sample 01Y-140_—FujiFilm film (LLW 0.5–2.5 MPa).

**Figure 22:**
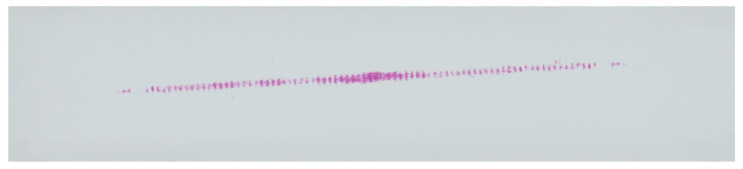
Sample 02Y-140—Sensorprod film (SPF-D 2.413–9.652 MPa).

The study included five measurements for each pressure measurement film. Detailed analytical results for the samples 01Y-140_ and 02Y-140_ are shown below. Both samples were obtained by loading the gear in the same position with the same mass of 18 kg, which was suspended from the last hole of the test stand arm. The basic characteristics of the sample before preprocessing are shown in Figure 20. In turn, Figure 21 and Figure 22 show the test samples already preprocessed.

**Figure 23:**
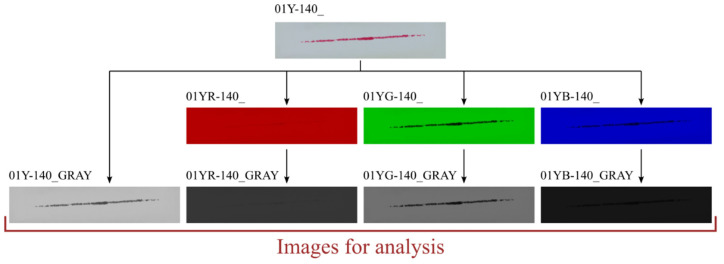
Sample 01Y-140_—images for analysis.

**Figure 24:**
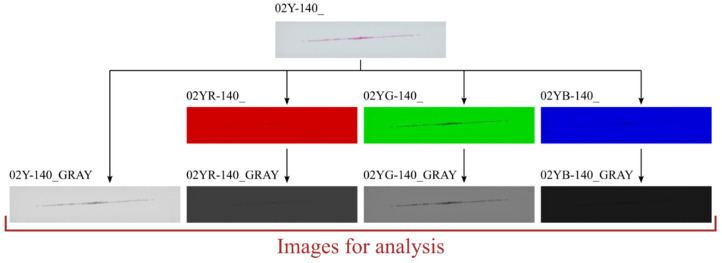
Sample 02Y-140_—images for analysis.

The contact pattern reflects the surface structure of the tooth side. The grooves seen in the above figures are due to 3D printing technology and, more specifically, to the thickness of the layers when 3D-printing the gear models. For each sample, three derivative samples were determined for each channel from the RGB color space, and so obtained, yielded four images (which were then converted to grayscale) per sample (Figure 23 and Figure 24).

**Figure 25:**
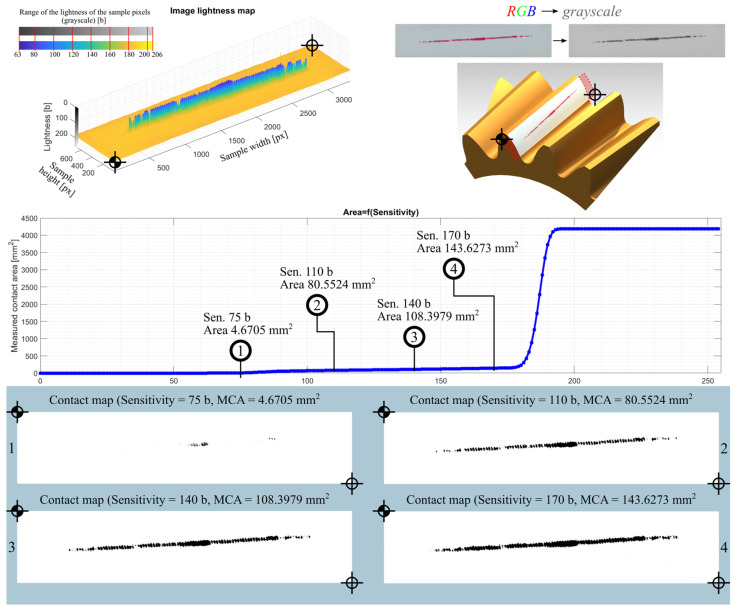
Report for sample 01Y-140_ (RGB → GRAY)—RGB image converted to grayscale.

**Figure 26:**
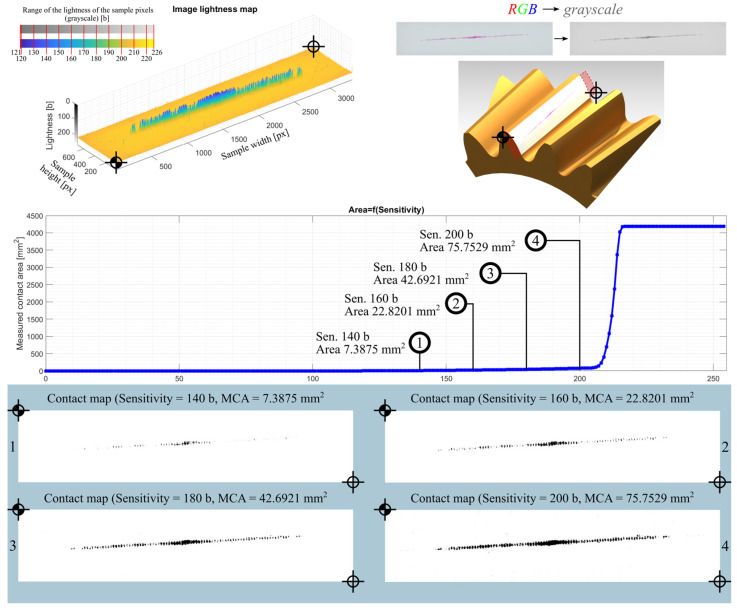
Report for sample 02Y-140_ (RGB → GRAY)– RGB image converted to grayscale.

**Figure A1:**
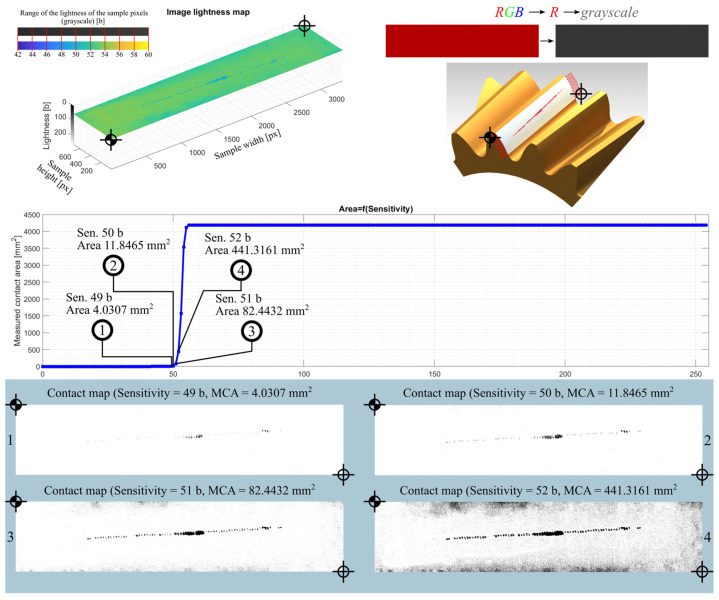
Report for sample 01Y-140_ (RGB → R → GRAY)—red channel image converted to grayscale.

**Figure A2:**
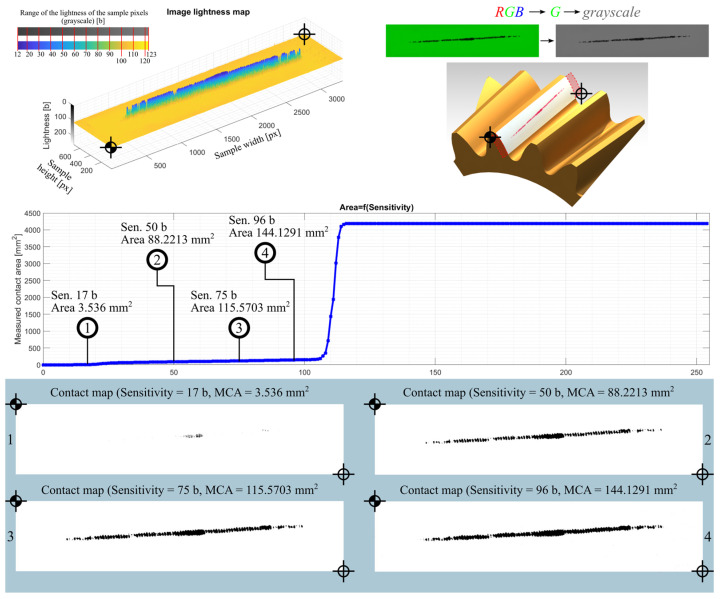
Report for sample 01Y-140_ (RGB → G → GRAY)—green channel image converted to grayscale.

**Figure A3:**
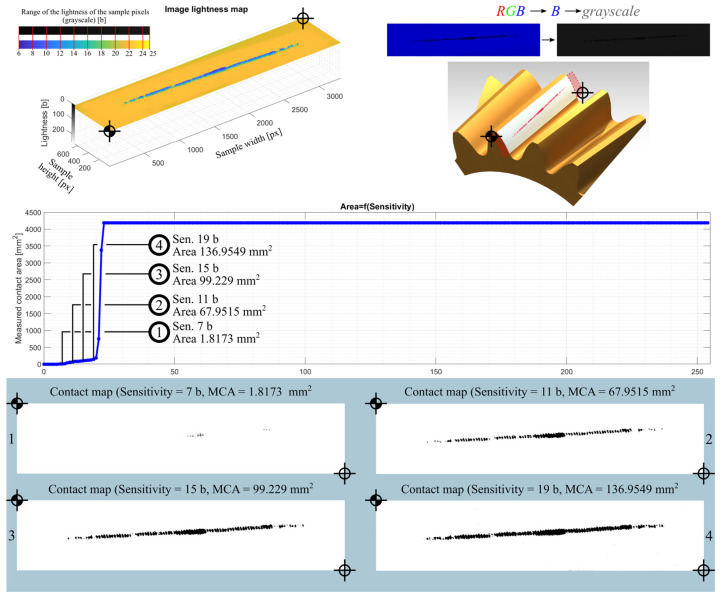
Report for sample 01Y-140_ (RGB → B → GRAY)—blue channel image converted to grayscale.

**Figure A4:**
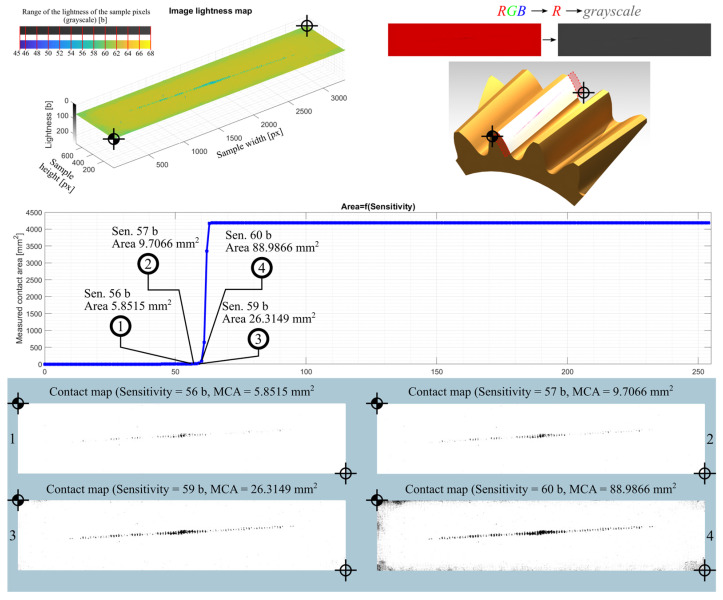
Report for sample 02Y-140_ (RGB → R → GRAY)—red channel image converted to grayscale.

**Figure A5:**
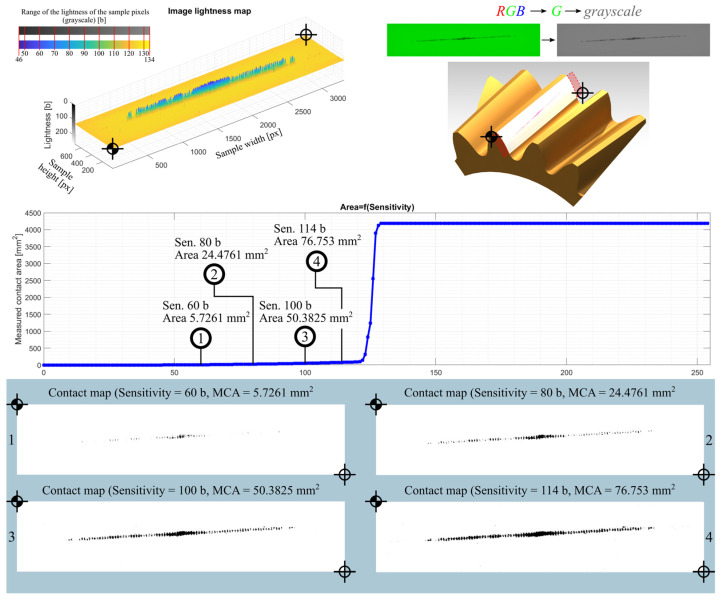
Report for sample 02Y-140_ (RGB → G → GRAY)—green channel image converted to grayscale.

**Figure A6:**
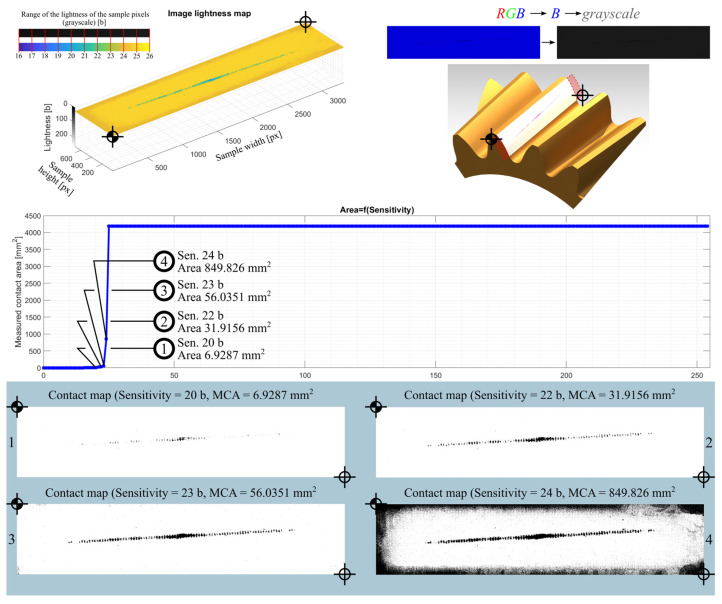
Report for sample 02Y-140_ (RGB → B → GRAY)—blue channel image converted to grayscale.

Reports of the results of the analysis of individual images, i.e., the lightness map of the sample, the superimposition of the sample image as a tooth-side texture on the 3D CAD model of the gear, the relationship between the *Sensitivity* parameter and the measured area of the contact trace, and the determined contact maps using the author’s program for four values of the *Sensitivity* parameter are shown in the figures below. Here, Figure 25 and Table 5 are for sample 01Y-140_ and are reports on the images (after conversion to grayscale) of the RGB, R channel, G channel, and B channel (Appendix B Figure A1, Figure A2 and Figure A3). Similarly, Figure 26, Table 6, and Appendix B Figure A4, Figure A5 and Figure A6 are for sample 02Y-140_.

Analyzing Figure 25, Figure 26, Figure A1, Figure A2, Figure A3, Figure A4, Figure A5 and Figure A6, it can be seen that for each sample and channel, there is a limiting value of the *Sensitivity* parameter LVS, above which there is a sharp increase in the measured area of the contact pattern. The limiting value of the *Sensitivity* parameter depends on, i.a., the manufacturer of the sheet, the measurement range of the sheet, and the humidity and temperature of the air in the laboratory during the measurement [58].

**Figure 27:**
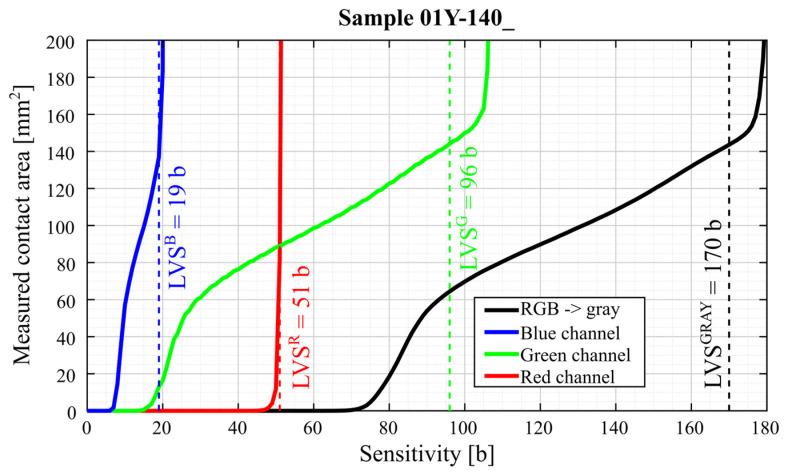

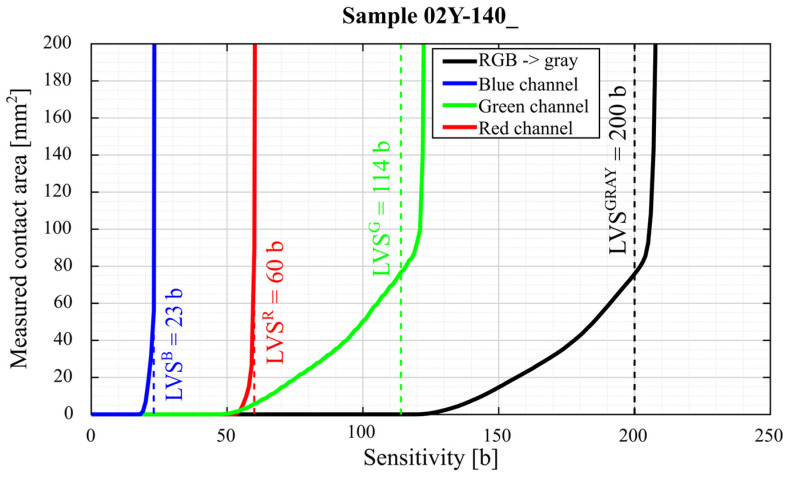
Comparison of curves representing the relationship between the *Sensitivity* parameter and the measured contact area using the FujiFilm film (LLW 0.5–2.5 MPa)—top—and Sensorprod (SPF-D 2.413–9.652 MPa)—bottom, where LVS—limit value of the *Sensitivity* parameter for the sample and specific channel (GRAY—grayscale, R—red, G—green, B—blue).

Figure 27 compares the analytical results of samples 01Y-140_ (top) and 01Y-140_ (bottom) with each other. To better show the nature of the variation of the individual curves for both samples, the range of variation of the *Sensitivity* parameter was limited.

As a result of the analysis, it was assumed that for the sample 01Y-140_ (RGB → GRAY), the limit value of the *Sensitivity* parameter is $LVS^{GRAY}=170\;b$. For this value of this parameter, the measured contact pattern area is equal to 143.6273 mm^2^. Due to the fact that the color sample 02Y-140_ (RGB → GRAY) is lighter (both the sheet and contact pattern), the *Sensitivity* parameter limit for this sample is higher and is equal to $\;LVS^{GRAY}=200\;b$. The measured contact pattern of the 02Y-140_ sample for the limiting lightness is equal to 75.7529 mm^2^.

LVS limit values were determined based on the analysis of the generated contact maps. The main criterion was the number of artifacts (misidentified contact pixels) located in areas of the sample, which were located far away from the area of high-color intensity coloring the sample. For a better comparison of the obtained results, fixed LVS values were assumed for the specific type of sheet and the analyzed channel (gray, red green, and blue). For more accurate results, it is recommended to individually select the values of limits. This process can be assisted by the use of AI algorithms, as described in the works of [67,68]. Contact maps and data generated using the programs described in Appendix A can be used to teach these algorithms. Comparison of the CAD, FEM, and experimental results is presented in Table 7.

**Figure 28:**
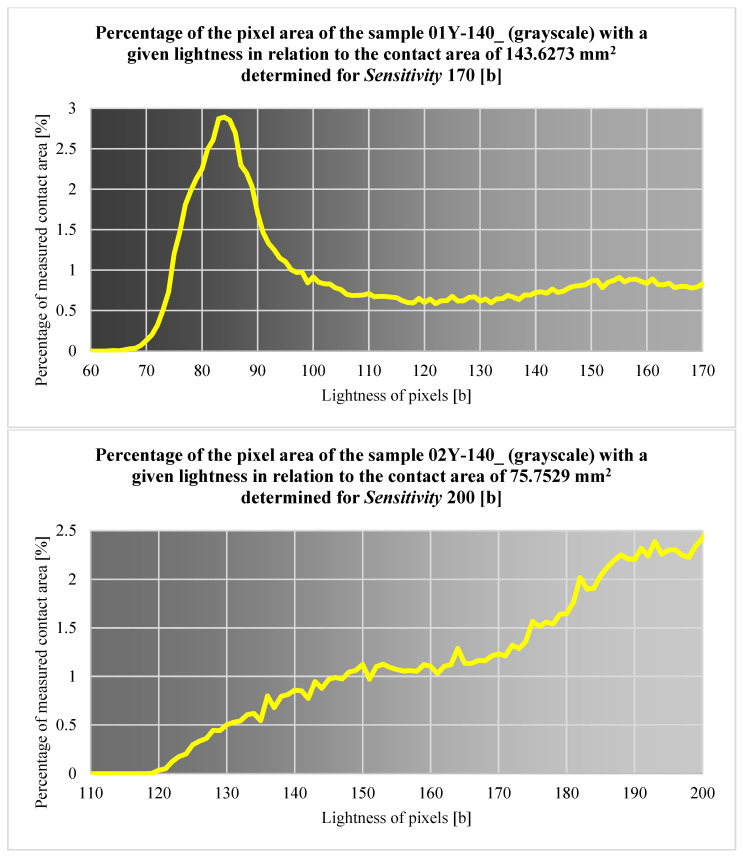
Percentage of measured area of the pixel of the contact pattern with a given lightness in relation to the total area of the contact pattern (the background of the charts corresponds to the lightness of the pixels).

The plots shown in Figure 28 illustrate the percentage of the total area of pixels of a particular lightness in relation to the total area of the sample. The graph at the top is for sample 01Y-140_ (FujiFilm), while the bottom graph is for sample 02Y-140_ (Sensorprod).

Analyzing the charts above, it can be seen that for film manufactured by FujiFilm, there is a clear peak in the percentage of pixels with lightness in the 75 to 90 b range. This increase is due to, i.a., the measurement range of the sheet. The pressure of the teeth caused by the load on the gears causes pressure near or above the measurement range of the LLW sheets in the contact zone. This causes intense discoloration of these pixels. Pixels in this range account for approximately 35.5% of the total contact area. On the other hand, in the case of SPF-D sheets designed to measure higher pressures, an increase in the proportion of the area of lighter pixels (range from 170 to 200 b) can be seen. These pixels amount to about 59.5% of the total contact area. In addition, the course of the curve for this sheet has an increasing character, where in the case of the LLW sheet, once reached, the percentage of lighter pixels was similar.

Based on the above considerations, it was concluded that when using pressure measurement film to measure the area of the contact pattern, the sheets should be selected so that their measurement range guarantees the largest possible share of the ‘darker pixels’ area in the total area of the contact pattern. This will make it easier to detect the edges of the contact area outline and reduce the *Sensitivity* parameter range. This can be achieved by using sheets designed to measure very small or low pressures, such as Extreme Low Pressure (4LW) for pressures 0.05–0.2 MPa or its equivalent manufactured by Sensorprod (SPF-A). It should be noted, however, that the use of highly sensitive pressure measurement films can cause significant problems, first of all, difficulties in mounting such sheets (especially on small teeth). These difficulties are due to the fact that such films are very easy to discolor when they are attached to the gear teeth, resulting in the tearing of some of the active substance capsules, even before the measurement begins.

**Figure 29:**
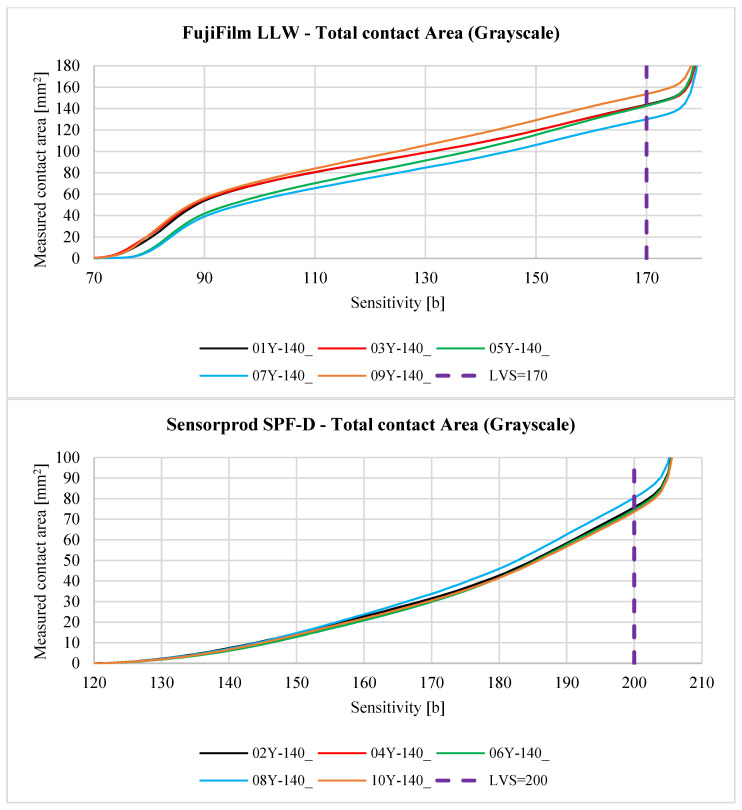
The relationship between the *Sensitivity* parameter value and the measured contact area for the grayscale.

**Figure 30:**
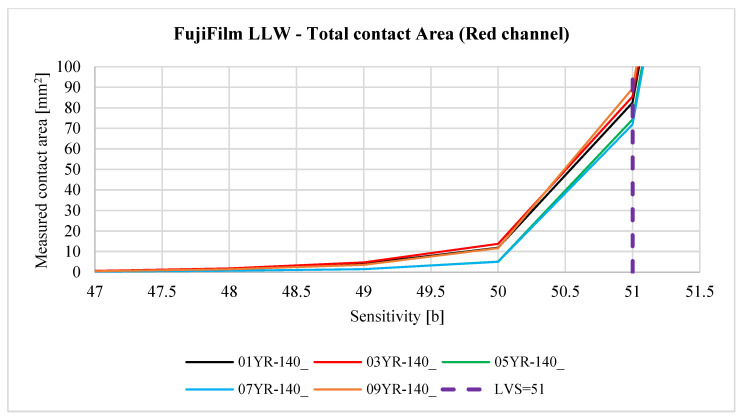

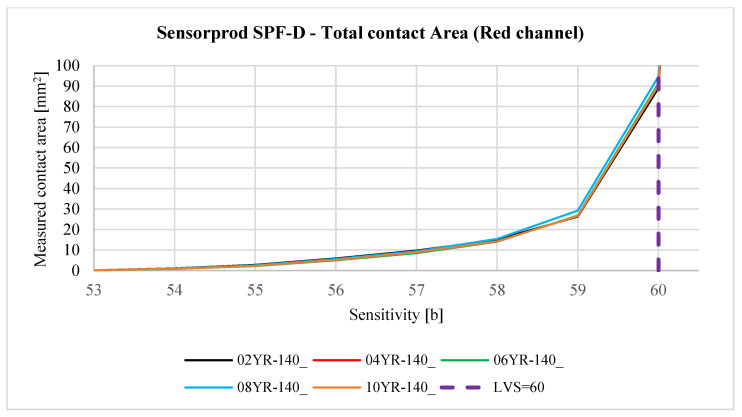
The relationship between the *Sensitivity* parameter value and the measured contact area for the red channel.

**Figure 31:**
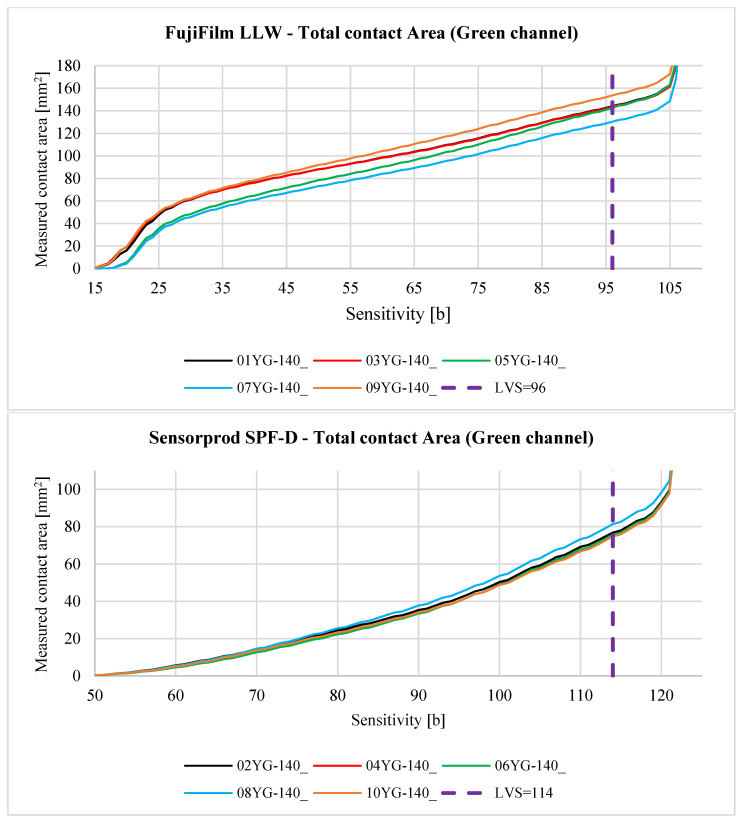
The relationship between the *Sensitivity* parameter value and the measured contact area for the green channel.

**Figure 32:**
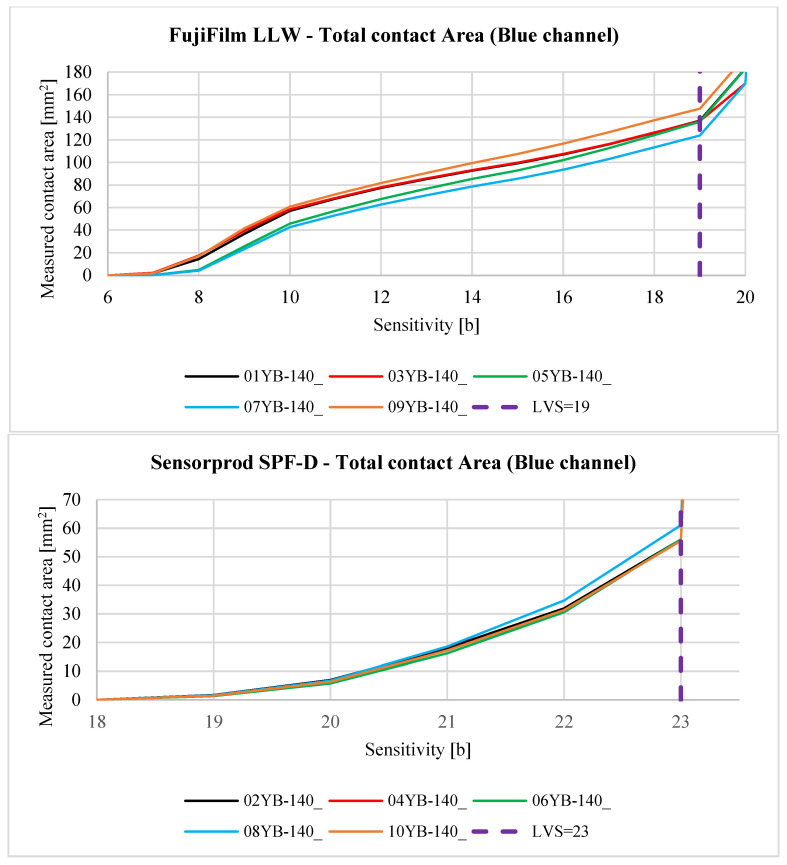
The relationship between the *Sensitivity* parameter value and the measured contact area for the blue channel.

**Figure 33:**
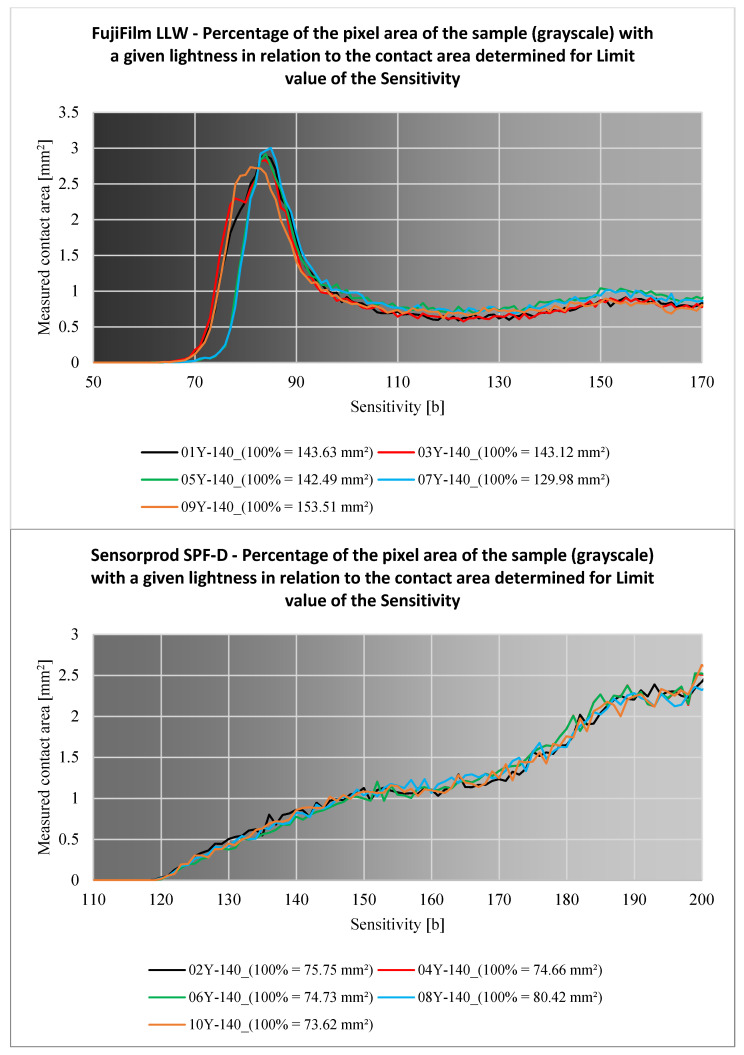
Percentage of the pixel area of the sample (grayscale) with a given lightness in relation to the contact area determined for the limit value of the *Sensitivity*.

Figure 29, Figure 30, Figure 31, Figure 32 and Figure 33 show the aggregate results for all obtained samples. The odd-numbered samples were obtained using pressure measurement films manufactured by FujiFilm, while the even-numbered samples involve sheets manufactured by Sensorprod.

**Figure 34:**
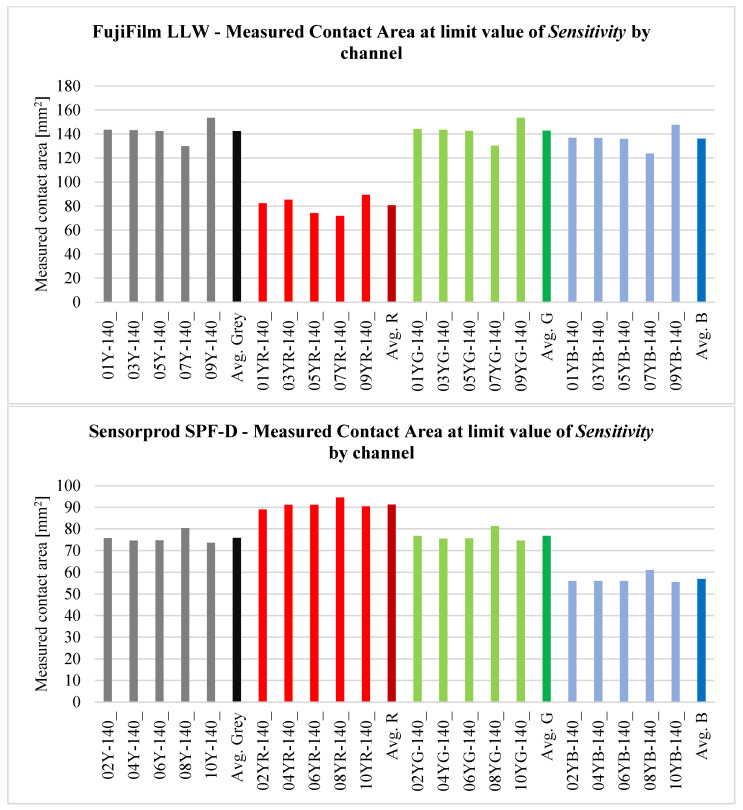
Measured contact area at limit value of *Sensitivity* by channel.

**Figure 35:**
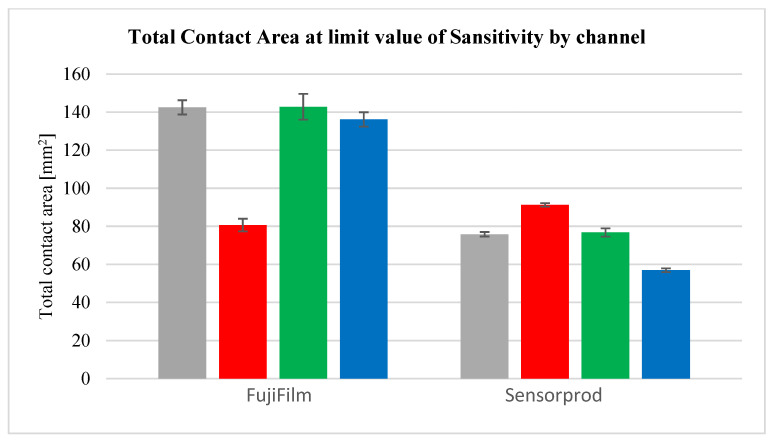
Measured contact area at limit value of *Sensitivity* by channels (grayscale, red, green, blue) —standard deviation.

After analyzing the curves shown in Figure 29, Figure 30, Figure 31, Figure 32 and Figure 33, it was found that the considerations presented after the analysis of samples 01Y-140_ and 02Y-140_ are also valid for the other samples. Figure 34 and Figure 35 shows the averaged values of measured contact areas obtained for limit values of *Sensitivity* LVS.

Statistical analysis of the graphs shown in Figure 34 show that for FujiFilm samples, the differences between the average values of contact pattern areas determined from the grayscale, green, and blue channels are comparable. It is recommended that for sheets from both manufacturers, contact pattern analysis should consider grayscale images or the green channel. In the case of FujiFilm sheets, blue channel analysis also yields similar results to grayscale or green channel images, but exceeding the LVS parameter limit results in a very large error compared to grayscale or green channel images.

#### 3.3.2. Image Data Processing—Contact Map Analysis

**Figure 36:**
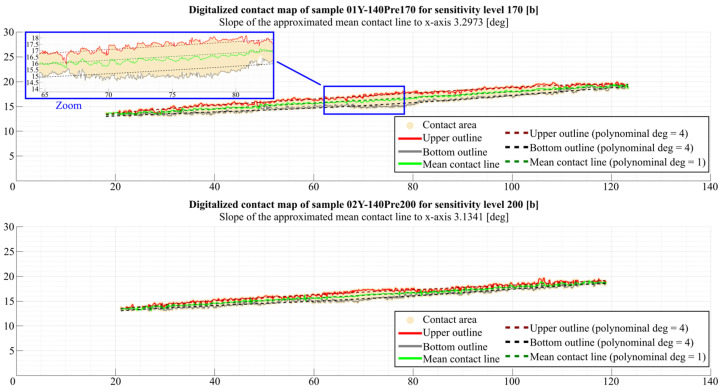
Curves describing the outline of the contact trace envelope and the average line of the contact pattern obtained from the analysis of the sample 01Y-140_ contact map obtained for the parameter $Sensitivity=170\;\left[b\right]$ (top) and the sample 02Y-140 contact map obtained for the parameter $Sensitivity=200\;\left[b\right]$ (bottom).

This section of the article presents the results of contact map analyses using the program described earlier. As with the analyses for measuring the area of the momentary contact pattern, the article presents the results of the analyses carried out for samples 01Y-140_ and 02Y-140_ (Figure 36). It is worth mentioning here that the aforementioned contact map was put through postprocessing in external software in order to remove artifacts that could disturb the program and negatively affect the obtained results. In addition, it should be noted that the results obtained are significantly influenced, among other things, by the denoising algorithm used and the degrees of polynomials used for approximation. In order to increase the efficiency of the denoising process, it is recommended to denoise the image obtained for the highest analyzed value of the *Sensitivity* parameter and then use it as a mask (whose black pixels are 100% transparent), which can be applied directly to the other images. Thanks to this way of denoising, it is possible to avoid deleting areas of the contact map, which at small values of the *Sensitivity* parameter are single pixels. This article presents results obtained using polynomials of degree 4 (for the upper and bottom outlines) and 1 (for the mean contact line). In addition, the contact maps analyzed were determined for grayscale images.

**Figure 37:**
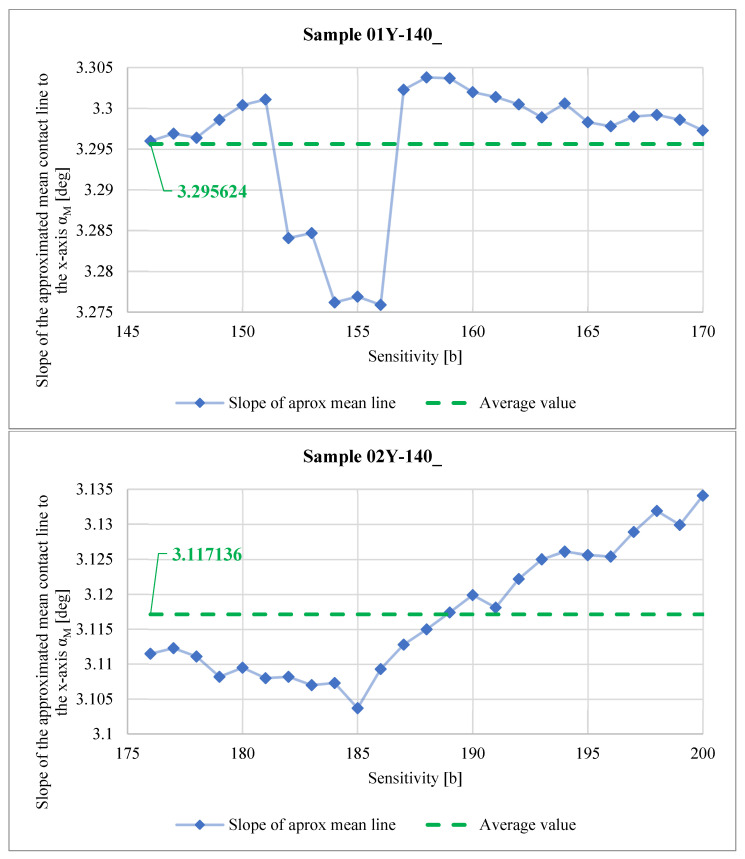
Slope of the approximated mean contact line to the x-axis $\alpha_{M}$.

Figure 37 shows how the slope of the approximated mean contact line to the x-axis $\alpha_{M}$ calculated using Equation (1) for samples 01Y-140_ (top) and 01Y-140_ (bottom) varied with the change in the *Sensitivity* parameter.

Analyzing the graphs shown in Figure 37, it can be seen that near the limiting values of the *Sensitivity* parameter (160–170 b) for sample 01Y-140_, the values of the $\alpha_{M}$ angle stabilize near the value of 3.3°, while for sample 02Y-140_, this angle, determined also for the last 10 values of the *Sensitivity* parameter (190–200 b), clearly increases relative to the average value. This is due to the phenomenon described in the commentary to the graphs shown in Figure 28.

**Figure 38:**
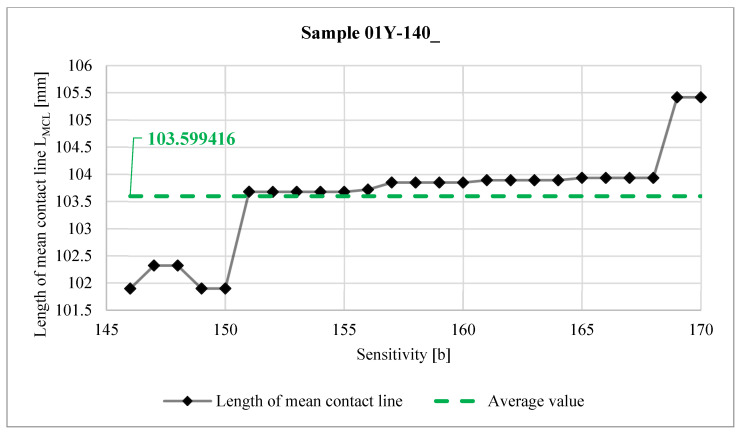

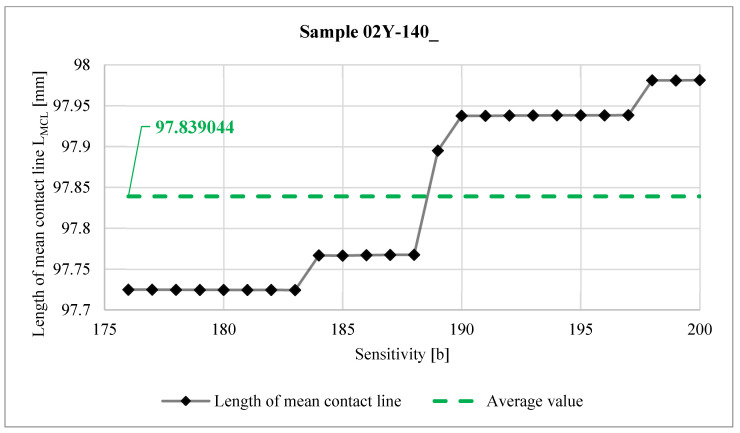
Length of mean contact line $L_{MCL}$.

Figure 38 shows the relationship between the *Sensitivity* parameter and the length of the mean contact line $\;L_{MCL}$ determined from Equation (2).

The differences in the lengths of the measured $L_{MCL}$ values are small, both within a given sample and with respect to the LLW to SPF-D sheets. The difference between the average $L_{MCL}$ length values obtained for both types of sheets is about 5.6% with respect to the average $L_{MCL}$ value for the LLW sample.

**Figure 39:**
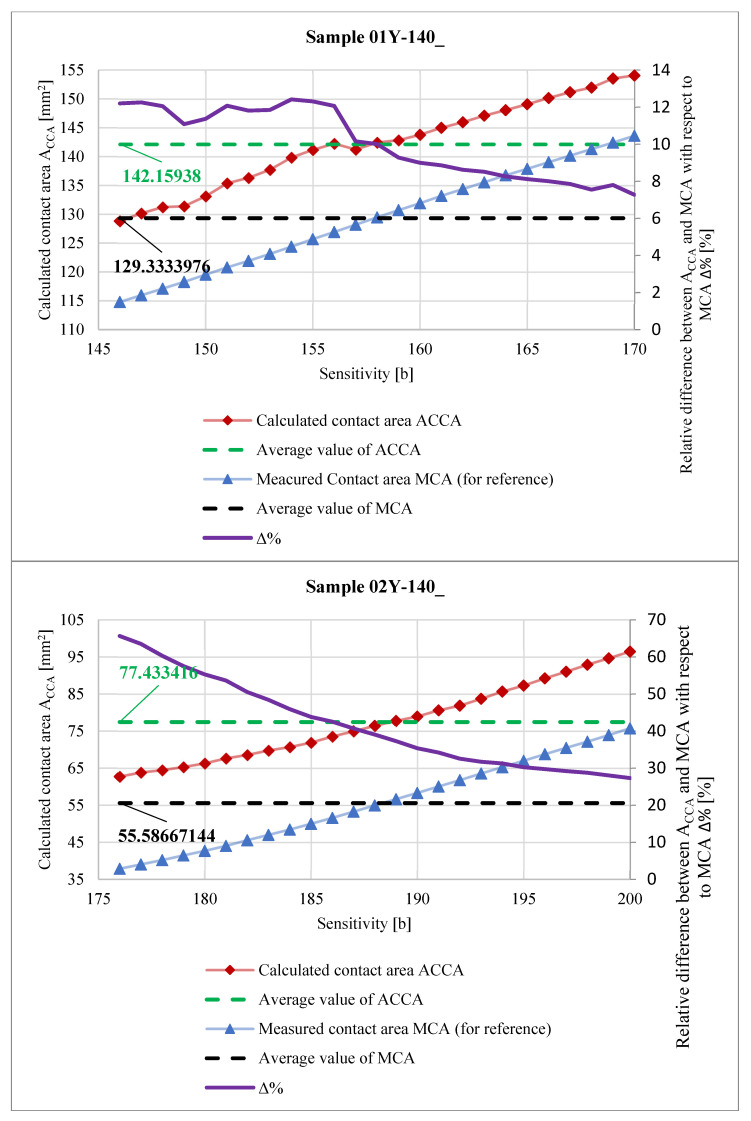
Calculated contact area $A_{CCA}$.

In Figure 39, the relationship between the calculated areas of the contact pattern $A_{CCA}$ determined by Equation (3) is shown. For a better comparison, a curve showing the relationship between the measured contact area MCA and the *Sensitivity* parameter is also added. The vertical axis on the right side of the graph relates to the purple curve, describing how the difference between areas $A_{CCA}$ and MCA with respect to area MCA changes (in percentage terms) as the *Sensitivity* parameter changes. This difference is described by the ∆% parameter calculated from Equation (4).

(4) $$∆\%\left(Sensitivity\right)=\frac{A_{CCA}\left(Sensitivity\right)-MCA\left(Sensitivity\right)}{MCA\left(Sensitivity\right)}\cdot 100\%\;\left[\%\right].$$

The differences between the values of areas $A_{CCA}$ and MCA (Figure 39) are mainly due to the fact that, in the case of meshing of the gears produced by incremental methods, there are small grooves on the tooth flank surface. These grooves are visible as unpressed areas that provide a striped character to the contact pattern. The wider these grooves are, the greater the differences will occur between the values of areas $A_{CCA}$ and MCA. The variances of the ∆% parameter are greater for SPF-D sheets and reach up to about 65%. This is mainly due to the measurement range of these pressure measurement films. Calculation of the contact pattern area as a result of integration of curves describing the upper and lower contours of the contact pattern allows one to partially compensate for errors resulting from the structure of gears produced by incremental methods and poor selection of pressure-measuring films; however, the degree of this compensation is an open question and may be the subject of further research.

**Figure 40:**
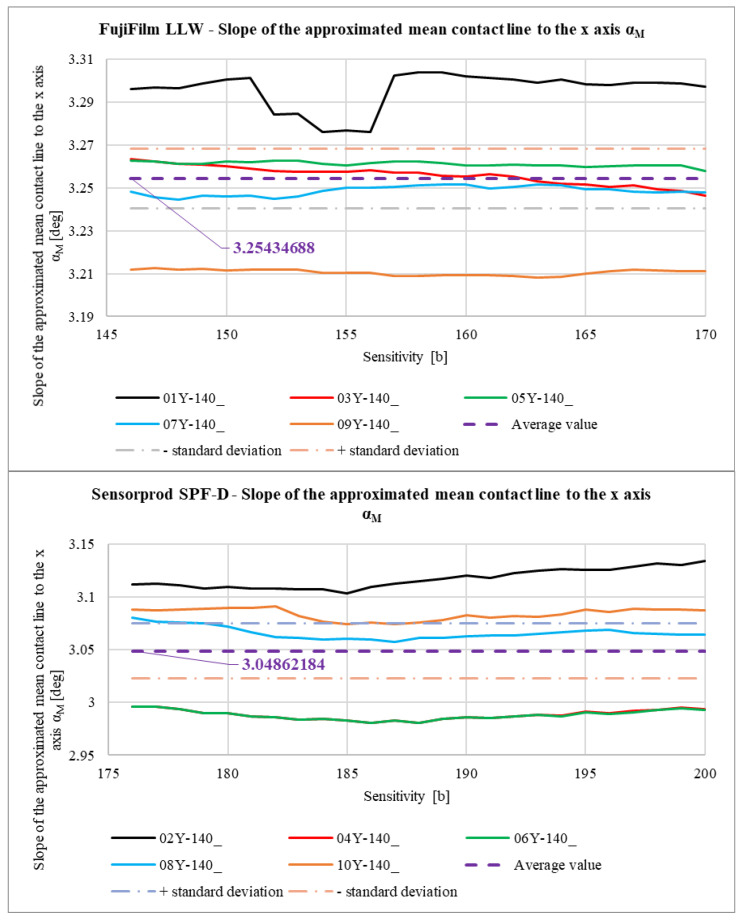
Comparison of the slope of the approximated mean contact line to the x-axis $\alpha_{M}$.

**Figure 41:**
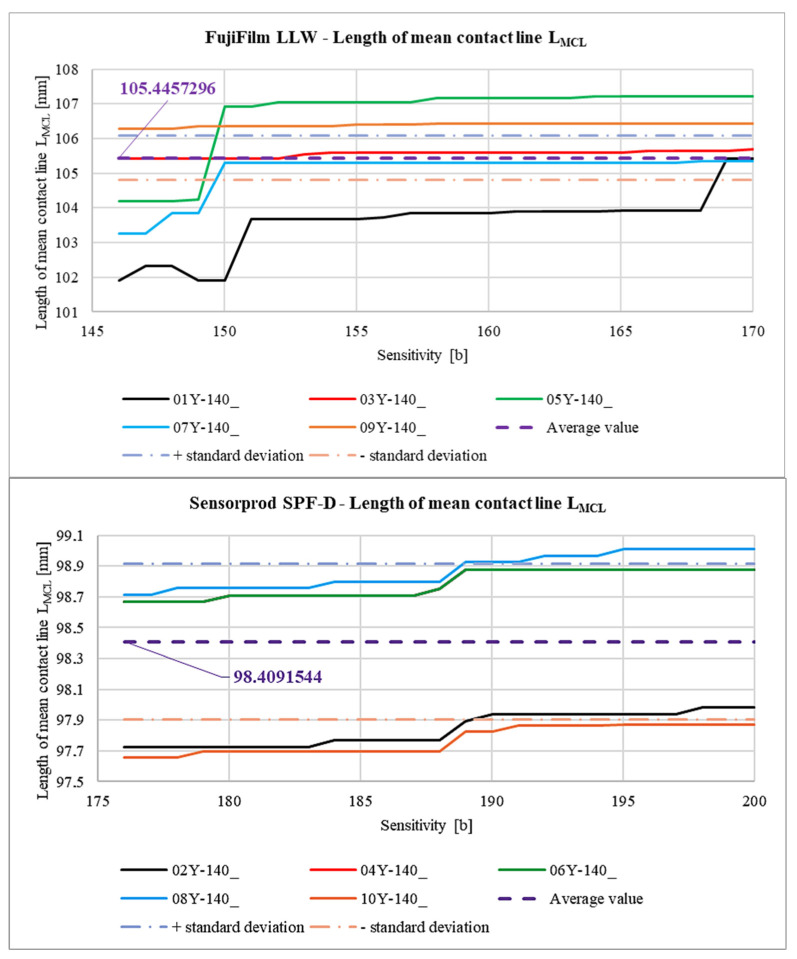
Comparison of the length of mean contact line $\;L_{MCL}$.

**Figure 42:**
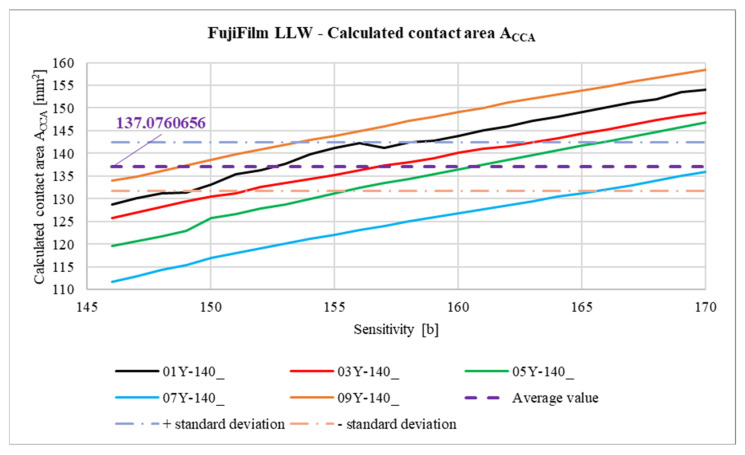

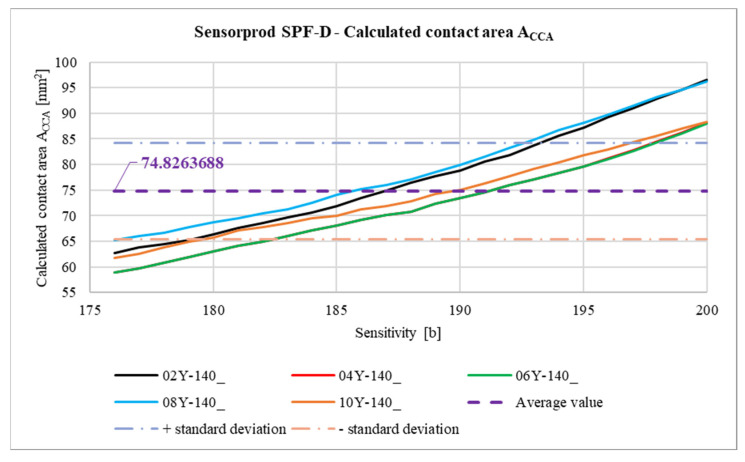
Comparison of the calculated contact area $A_{CCA}$.

**Figure 43:**
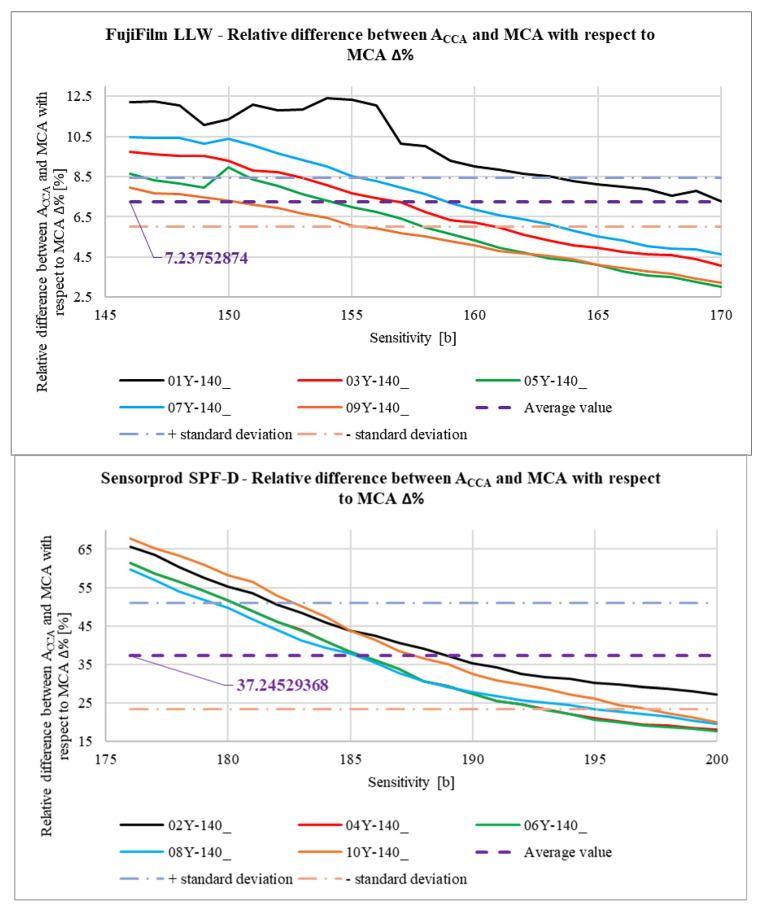
Comparison of ∆% parameter.

In Figure 40, Figure 41, Figure 42 and Figure 43, average results for all 10 samples are presented to compare the results obtained for the LLW and SPF-D sheets. It is worth mentioning here that the average values were calculated as arithmetic averages of all obtained for the films of a given manufacturer, that is, 125 values for each parameter marked with a violet dashed line.

As a result of the analysis of the graphs shown in Figure 40, Figure 41, Figure 42 and Figure 43, it was found that the observations made on the basis of Figure 37, Figure 38 and Figure 39 are also valid for a larger number of measurements, and the characters of the curves corresponding to each other are similar.

## 4. Conclusions

The developed method for measuring the contact pattern using a pressure film is effective. As a result of the analysis of the results obtained through theoretical (TCA analysis in CAD and LTCA analysis using FEM) and experimental (LTCA) studies, the following was found:

• The results of contact pattern investigations using pressure measurement films are consistent with the analytical and numerical study results.
• The values obtained for the slope angles of the average contact line determined for both samples are comparable (the relative error is approximately 5%).
• The pressure measurement films used in the study can be successfully used to study the contact pattern of cylindrical gears, including those with a sinusoidal profile.
• The method can be used under workshop conditions (without the need for computer analysis of sample scans) to quickly determine the correctness of the gearbox assembly.
• The results of contact trace investigations using pressure-sensitive films are consistent with the analytical and numerical study results.
• The developed algorithm for determining the contact pattern with the use of an office optical scanner can replace the need to purchase a dedicated scanner and software from film manufacturers for pressure measurement.
• Sensorprod films are lighter, which can negatively affect the accuracy of the measurement, as it is easier to lose image detail in the scanning process. In addition, due to the higher lightness of the contact pattern (from about 140 to 220 bits in grayscale), it is much more difficult to distinguish the noise and fine contaminants visible on the sample scan from the actual contact pattern.

Further research can be aimed at increasing the efficiency of testing using machine learning methods. The data obtained using the described method can provide a dataset to teach artificial intelligence models.

Further research is planned to apply the presented methods to other types of gears, tooth profiles, and gear materials. Next steps will be also concerned with determining the effect of assembly errors on the position and shape of the contact pattern.

## Figures and Tables

**Table 1:** Data of analyzed gear pair.

Parameter	Pinion	Gear
Normal module	$m_{n}=15\;mm$	
Number of teeth	$z_{1}=21\left[-\right]$	$z_{2}=21\left[-\right]$
Normal pressure angle	$\alpha_{n}=20^{o}$	
Helix angle	$\beta =10^{o}$	
Face width	b=125 mm	
Center distance	a=315 mm	
Addendum	$h_{a}=15\;mm$	
Dedendum	$h_{f}=18.25\;mm$	
Circumferential clearance	$j_{t}=0.75\;mm$	

**Table 2:** FujiFilm Prescale Super Low Pressure (LLW) parameters [58].

Manufacturer’s Label	Value
Measurement Range	0.5–2.5 MPa
Operating Temperature	20–35 °C
Humidity Range	35–80% RH
Accuracy	±10% or less (measured with a densitometer at 23 °C, 65% RH)
Type	Two-sheet type

**Table 3:** Sensorprod SPF-D parameters [65].

Manufacturer’s Label	Value
Measurement Range	350–1400 (2.413–9.652 MPa)
Operating Temperature	5–35 °C (41–95 °F)
Humidity Range	10–90 %RH
Gauge (Thickness)	0.19 mm (7.5 mils)
Spatial Resolution	2.6 µm
Accuracy	±10%
Shelf Life	1 Year
Type	Two-sheet type

**Table 4:** RDG 720 material parameters [66].

Parameter	Value
Modulus of elasticity	2000
Ultimate tensile strength	50
Density	1.185
Elongation at break	15
Hardness Shore D	8–3

**Table 5:** Measured contact area (FujiFilm LLW).

Sample	Sensitivity [b]	Measured Contact Area $\left[mm^{2}\right]$
01Y-104_	75	4.6705
	110	80.5524
	140	108.3979
	170	143.6273
01YR-104_	49	4.0307
	50	11.8465
	51	82.4432
	52	441.3161
01YG-104_	17	3.5360
	50	88.2213
	75	115.5703
	96	144.1291
01YB-104_	7	1.8173
	11	67.9515
	15	99.2290
	19	136.9549

**Table 6:** Measured contact area (Sensorprod SPF-D).

Sample	Sensitivity [b]	Measured Contact Area $\left[mm^{2}\right]$
02Y-104_	140	7.3875
	160	22.8201
	180	42.6921
	200	75.7529
02YR-104_	56	5.8515
	57	9.7066
	59	26.3149
	60	88.9866
02YG-104_	60	5.7261
	80	34.4761
	100	50.3825
	114	76.7530
02YG-104_	20	6.9287
	22	31.9156
	23	56.0351
	24	849.8260

**Table 7:** Comparison of results obtained by CAD-based, FEM-based, and experimental methods.

CAD Method	FEM Method	Experimental Method
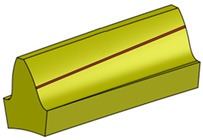 Contact pattern area $\approx 161\;\left[mm^{2}\right]$	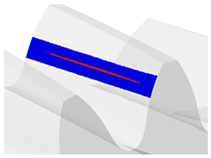 Contact pattern area $\approx 137\;\left[mm^{2}\right]$	FujiFilm LLW 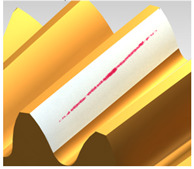 Sample 01Y-140_ (at sensitivity 170 b) 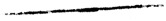 Contact pattern area $\approx 144\;\left[mm^{2}\right]$ Sensorprod SPF-D 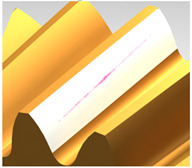 Sample 02Y-140_ (at sensitivity 200 b) 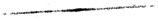 Contact pattern area $\approx 76\;\left[mm^{2}\right]$

## Data Availability

The original contributions presented in this study are included in the article. Further inquiries can be directed to the corresponding authors.

## Appendix A

A code fragment of the *ContactArea* function responsible for detecting contact pixels and generating a contact map is presented:

*01*	*for* *i = 1:1:size (ImgeGrayscale,1)* *% Loop selecting horizontal line of image*
*02*	
*03*	*for* *j = 1:1:size (ImgeGrayscale,2)* *% Loop selecting vertical line of image*
*04*	
*05*	*% Checking pixel lightness*
*06*	*if* *ImgeGrayscale (i, j) <= Sensitivity* *% Condition fulfilled*
*07*	
*08*	*MCA = MCA + ppa;* *% Increase the contact area*
*09*	
*10*	*ContactMap (i, j) = uint8(0);* *% Color pixel black (contact detected)*
*11*	
*12*	*else % Condition not fulfilled*
*13*	
*14*	*ContactMap (i, j) = uint8(255);* *% Color pixel white (contact not detected)*
*15*	
*16*	*end*
*17*	
*18*	*end*
*19*	
*20*	*end*
